# Sense of Accomplishment Is Modulated by a Proper Level of Instruction and Represented in the Brain Reward System

**DOI:** 10.1371/journal.pone.0168661

**Published:** 2017-01-04

**Authors:** Tomoya Nakai, Hironori Nakatani, Chihiro Hosoda, Yulri Nonaka, Kazuo Okanoya

**Affiliations:** 1 Graduate School of Arts and Sciences, The University of Tokyo, Tokyo, Japan; 2 Japan Society for the Promotion of Science, Tokyo, Japan; 3 Center for Evolutionary Cognitive Science, The University of Tokyo, Tokyo, Japan; 4 PRESTO, Japan Science and Technology Agency (JST), Tokyo, Japan; 5 Riken Brain Science Institute, Saitama, Japan; University of Pennsylvania, UNITED STATES

## Abstract

Problem-solving can be facilitated with instructions or hints, which provide information about given problems. The proper amount of instruction that should be provided for learners is controversial. Research shows that tasks with intermediate difficulty induce the largest sense of accomplishment (SA), leading to an intrinsic motivation for learning. To investigate the effect of instructions, we prepared three instruction levels (No hint, Indirect hint, and Direct hint) for the same insight-problem types. We hypothesized that indirect instructions impose intermediate difficulty for each individual, thereby inducing the greatest SA per person. Based on previous neuroimaging studies that showed involvement of the bilateral caudate in learning and motivation, we expected SA to be processed in this reward system. We recruited twenty-one participants, and investigated neural activations during problem solving by functional magnetic resonance imaging (fMRI). We confirmed that the Indirect hint, which imposed intermediate difficulty, induced the largest SA among the three instruction types. Using fMRI, we showed that activations in the bilateral caudate and anterior cingulate cortex (ACC) were significantly modulated by SA. In the bilateral caudate, the indirect hint induced the largest activation, while the ACC seemed to reflect the difference between correct and incorrect trials. Importantly, such activation pattern was independent of notations (number or letter). Our results indicate that SA is represented in the reward system, and that the Indirect instruction effectively induces such sensation.

## Introduction

Within the field of education science, there has been many discussions regarding the level of instruction that should be provided for students [1–3]. Proper instructions may change the subjective difficulty, even within the same problem, thereby encouraging students to be successful in problem solving. One such putative instruction strategy is the discovery learning strategy [2, 4], in which minimal instruction may encourage students to discover knowledge by themselves, rendering them intrinsically motivated to learn. Conversely, several studies have claimed that direct instruction, which provides sufficient information regarding concepts and procedures necessary for problem solving, is more efficient for learning [3, 5]. Although many discussions have been focused on these two extremes, it is reasonable to consider a “guided discovery” strategy with indirect instructions, which lies between pure discovery (with minimal instructions) and direct instructions [1, 6].

It has been proposed that tasks with intermediate difficulty, which are not too easy, nor impossibly hard, will induce the largest satisfaction for children after solving the problem [7–8]. One theoretical model proposed that satisfaction induced by solving problems, or sense of accomplishment (SA), enhances motivation for learning [9]. Indeed, another study also indicated that the most obvious and ubiquitous source of intrinsic motivation for learning would be to offer challenging activities to individuals at an intermediate difficulty for them [10]. It is likely that individuals do not feel SA under the direct instruction because the task becomes too easy for them, and that the minimal instruction is ineffective due to a lower successful rate. We hypothesized that even when solving the same problem participants would feel the greatest SA with indirect instruction, which may impose intermediate difficulty for them.

The application of neuroscience to education has recently attracted a wide variety of attention [11–12]. Neuroimaging techniques such as functional magnetic resonance imaging (fMRI) would help us to clarify learning-related cognitive functions at a more detailed level comparing to the behavioural methods alone [12]. In the present study, we sought to examine the neural basis of SA. In particular, we focused on the bilateral caudate, and anterior cingulate cortex (ACC). Recent neuroimaging studies revealed the involvement of the caudate, a part of the reward system, in processing of motivations [13–14]. The caudate has been also reportedly involved in reward anticipation and reward-based learning [15–16]. Consistent with human neuroimaging studies, activations in primate caudate neurons were modulated by motivational context [17]. As subparts of the striatum, we also examined activations in the ventral putamen and nucleus accumbens (NAcc), which were reportedly involved in the reward anticipation [18, 19]. However, we expect that the caudate is more related to SA, because SA may be relevant to the motivation-enhancing aspect in learning. Several studies have reported that the ACC is involved in processing emotional salience [20–21], and it is possible that emotional salience is tied up with the satisfaction of problem-solving as a secondary effect. We predicted that activations in the bilateral caudate would principally reflect SA induced by problem-solving.

**Fig 1:**
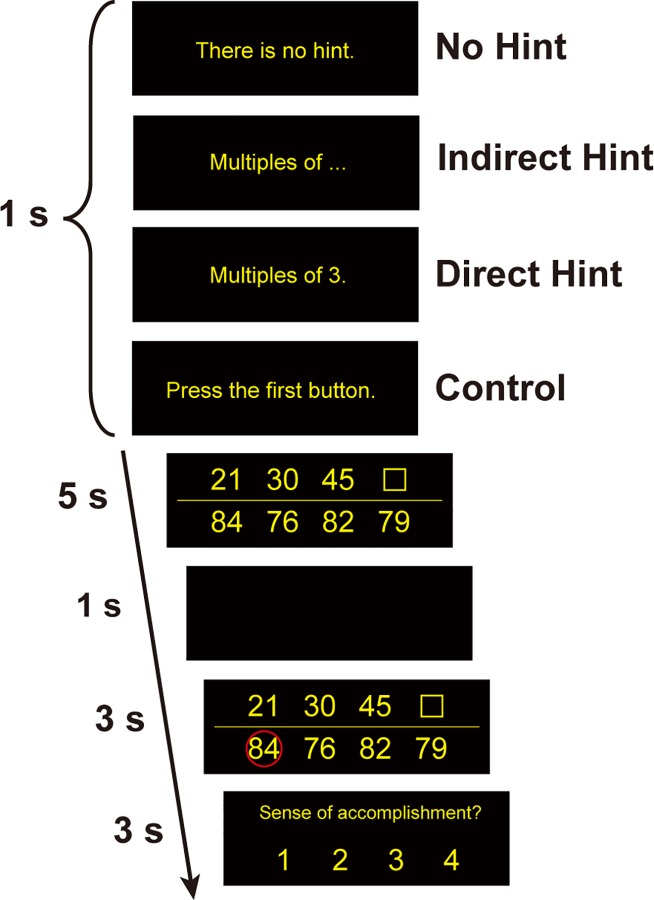
Experimental design. One of the four types of instructions was presented for 1 s, followed by the problem stimulus. Participants performed Number/Letter completion or button-press control tasks during the 5 s interval. After 1 s of a blank screen, 3 s of correct-answer feedback stimulus was presented. After the feedback stimuli, the participants rated their sense of accomplishment (SA) during the 3 s interval. Here we have shown an example of a Number notation task (translated in English).

To investigate the effects of instruction upon neural activations, we prepared three levels of instructions (No hint, Indirect hint, and Direct hint) for the same insight-problem types, and measured neural activations using fMRI (Fig 1). We enhanced the instruction effect by using insight-problem solving task which is difficult to solve without any instructions. To ensure that the instruction effect is not domain-specific, we created insight-problems with number and letter notations. We prepared a basic control with smaller SA by including a time-counting task that did not require problem solving (simply called “Control”). We first performed a whole-brain analysis to reveal brain regions where activations are modulated by SA. According to our hypothesis, we then examined signal changes in the anatomically defined ROIs of the bilateral caudate and ACC, but we also tested other functionally/anatomically defined ROIs for an exploratory purpose (see Methods for the detailed description). Our current results indicate that SA is represented in the reward system, and that the Indirect instruction effectively induces such sensation.

## Materials and Methods

### Participants

Twenty-one native Japanese speakers (14 males, aged 18–24 years) participated in the current experiment. All participants were healthy and right-handed (laterality quotient: 60–100), according to the Edinburgh inventory [22]. Prior to their participation in the study, written informed consent was obtained from all participants. This experiment was approved by the Ethics Committee of the University of Tokyo, Komaba.

### Stimuli and Tasks

The problem stimuli were composed of two arrays, where the upper array contained three symbols and a blank square, and the lower array contains four symbols. Sixty-four problem stimuli were created in total. To avoid memory-based interactions between the Direct condition and the other two instruction types, we used different problem stimuli sets in the Direct condition compared to the No hint and Indirect conditions. Since no memory-based interaction was expected, we used the same problem stimulus sets in the Direct and Control conditions. Although the problem stimuli were not common among three instruction levels, all problem stimuli were created based on the same set of rules. For the number notation, we used one-digit or two-digits of Arabic numbers. For the letter notation, Japanese letters (Hiragana) were used. Since Japanese participants learned serial, alphabet-like arrangements of those letters through their normal educational course, these letters were a perfect counterpart to numbers as fully familiarized sequential symbols for native Japanese participants.

We prepared four conditions (No hint, Indirect, Direct, and Control) for each of two symbol notations (Number and Letter; Fig 1 and S1 Fig). Each direct hint was constructed to give the participants full information regarding the procedures or concept that composed the number/letter array, which enabled the participants to solve the corresponding problems without considering any hidden rule. Indirect hints were systematically constructed by omitting one word from the corresponding direct hint. We chose to omit words that had parametric information (e.g., a base number “3” of multiples) necessary for solving the tasks, such that the participants had to find that parameter from various candidates. We made eight direct hint stimuli and corresponding indirect hint stimuli for each of two notations, and prepared four different problem stimuli depicted by each direct hint.

The participants were asked to perform a symbol (number or letter) completion task. In each trial, one of four condition instructions was given to the participants 1 s before the presentation of problem stimuli (e.g., No hint: “No Hint”, Indirect: “multiples of …,” Direct: “multiples of 3,” and Control: “Push the first button”). The participants selected a symbol from four candidates in the lower array to replace the blank square in the upper array, and pressed one of the four corresponding buttons (two buttons for the right hand, two for the left) as soon as possible within 5 s. We instructed the participants to press any one of four buttons even if they could not find an answer during this period, because we wanted to measure the reaction time in all of the trials for the fMRI analysis. In such case we allowed participants to press buttons after 5 s. For the example in Fig 1, three numbers were presented in the upper row (21, 30, 45). Since all of those numbers were multiples of 3, participants had to find the corresponding number (i.e., 84) which was also a multiple of 3, from the four candidates in the lower row (84, 76, 82, 79). They pressed the first button in this case. After 5 s, a black screen was presented for 1 s, followed by a correct-answer feedback stimulus for 3 s, where the correct symbol in the lower array was marked with a red circle. Finally, the participants rated SA that they felt for the task during the 3 s interval (1: “Strongly disagree”, 2: “Disagree”, 3: “Agree”, 4: “Strongly agree”). The next trial began after 1 s.

The fMRI experiment was composed of four scanning sessions, where each session consisted of 32 trials (four trials under each condition in each notation). Four conditions were randomly arranged as an event-related design. The total number of trials was 128, while the total number of problem stimuli was 64 (i.e., the same problem stimuli were used twice). At the end of each session, the accuracy of each condition in that session was displayed. To reduce the practice effect among stimuli, trials were counter-balanced across participants, and the order of trials was pseudorandomised under the following constraint: trials with the same problem stimuli were always arranged in the distant sessions (i.e., 1st and 3rd sessions, or 2nd and 4th sessions), and all trials in the same instruction type within a session were based on the different rules. After the fMRI experiment and outside the scanner, the participants were asked to assess the difficulty of all problem stimuli used in the fMRI experiment (i.e., intrinsic problem difficulty), in four-point Likert scale ranging from 1: “Very easy” to 4: “Very difficult.” by looking each problem stimulus with its direct hint. These questionnaires were composed of twelve questions with a seven-point Likert scale, ranging from 1: “Strongly disagree” to 7: “Strongly agree.”

The participants wore earplugs in the scanner. Stimuli were presented on a liquid-crystal display monitor (resolution: 1920 × 1080), so that participants viewed them through a mirror. For fixation, a small red cross was always shown at the centre of the screen. Reaction time (RT) was measured from the onset of the problem stimuli. The stimulus presentation and collection of behavioural data (accuracy and RT) were controlled using the Presentation software (Neurobehavioral Systems, Albany, CA).

### Behavioural Data Analyses

Correct/incorrect trials were evaluated by the response matching regardless of RTs, and trials with no response were treated as incorrect trials. In the present study, we evaluated the efficacy of instructions by overall results of problem-solving including both successful and unsuccessful cases, not only by the successful cases with limited numbers. Therefore, we included both correct and incorrect trials for the SA calculation. We also divided all trials (apart from those under the Control condition) into three difficulty levels (Hard, Medium, and Easy) according to the length of RTs (long, intermediate, and short, respectively) for each participant. 32 trials with the shortest RTs were categorised as Easy, 32 trials with the longest RTs were categorized as Hard, and remaining 32 trials were categorised as Medium, wherein the same trial was not always included in the same difficulty level for each participant. Trials with no response were included in the Hard level in this analysis.

### MRI Data Acquisition

The functional imaging was conducted on a 3.0 T scanner (MAGNETOM Prisma; Siemens, Erlangen, Germany) with a 20-channel head coil. We scanned 35 interleaved axial slices that were 3.2-mm thick with a 0.8 mm gap, parallel to the anterior and posterior commissure line, using a T2*-weighted gradient-echo echo-planar imaging (EPI) sequence [repetition time (TR) = 2.0 s, echo time (TE) = 30 ms, flip angle (FA) = 90°, field of view (FOV) = 192 × 192 mm^2^, resolution = 3 × 3 mm^2^]. In a single session, we obtained 242 volumes following four dummy images, which allowed for the rise of the MR signals. For anatomical reference, high-resolution T1-weighted images of the whole brain (176 sagittal slices, 1 × 1 × 1 mm^3^) were also acquired from all participants with a Magnetization Prepared Rapid Acquisition Gradient Echo sequence (MPRAGE, TR = 2000 ms, TE = 2.9 ms, FA = 9°, FOV = 256 × 256 mm^2^).

### fMRI Data Analyses

We performed fMRI data analyses using SPM12 statistical parametric mapping software (Wellcome Trust Centre for Neuroimaging, London, UK; http://www.fil.ion.ucl.ac.uk/spm/). The acquisition timing of each slice was corrected using the middle slice as a reference for the EPI data. We realigned the EPI data from multiple sessions to the mean image across all sessions. Each participant’s T1-weighted structural image was coregistered to the mean functional image generated during realignment, and then spatially normalized to the Montreal Neurological Institute (MNI) space with the new unified normalization-segmentation tool in SPM12. After spatial normalization, the resultant deformation field was applied to the realigned functional imaging data, resampled into 2 mm isotropic voxels. All normalized functional images were then smoothed using an isotropic Gaussian kernel of 8 mm full-width at half maximum. Low-frequency noise was removed by high-pass filtering at 1 / 128 Hz.

To separate the effect of reward anticipation and outcomes, we examined neural activations in both problem-solving and answer-feedback periods, by performing parametric modulation analyses of the fMRI data with two general linear models (GLM). Based on the previous studies of insight-problem solving [23–24], in the first GLM we used regressors composed of 3 s box-car functions, starting from 2 s before the button response for each trial (problem-solving period), convolved with a hemodynamic function. We selected this period to include the entire processes regarding SA and emotion related to the insight-problem solving. In the second GLM we used regressors composed of 3 s box-car function beginning with the onset of the correct-answer feedback stimulus (answer-feedback period). In both of the above GLMs, we merged all four conditions and two notations, and included SA as a parametric modulator for each participant. Since fMRI data were analysed based on the participants’ response, we excluded trials with no response from fMRI data analyses. A very small number of trials were excluded as no response trials (Table A in S1 File). The contrast image of positive modulation by SA was obtained for each participants, and was used for intersubject comparisons in the second-level analysis. The statistical threshold was set to *P* < 0.001 for the voxel level, with *P* < 0.05 for the cluster level [topological False-discovery rate (FDR) correction for multiple comparisons] across the whole brain [25]. We also reported some contrasts with uncorrected *P* < 0.001 which did not show significant activation with topological FDR correction, but might be suggestive for the interpretation of our data.

For the region of interest (ROI)-based beta estimate analysis, we made two additional GLMs with four conditions for both Number and Letter notations, in both problem-solving and answer-feedback periods in the same way as above, but without SA regressors. Based on our a priori interest in the regions related to the reward system and emotional saliency, we defined three anatomical ROIs for the bilateral caudate and ACC with the Automatic Anatomical Labeling atlas [26], two ROIs of 6 mm sphere from peak MNI coordinates of the left ventral putamen (–26, 8, –4) and right putamen (26, 6, –8) taken from a previous study of reward-based learning [18], two ROIs of 6 mm sphere from peak MNI coordinates of the left nucleus accumbens (NAcc) (-10, 10, -2) and right NAcc (10, 8, 0) taken from a previous meta-analysis study of the reward systems [19], and three functionally defined ROIs of the bilateral caudate and ACC from the contrast of positive modulation by SA. Since the anatomical ACC ROI covers large areas, we also examined two ROIs of 6 mm sphere from peak MNI coordinates of pregenual ACC (pACC, 2, 46, 20) and subcallosal ACC (sACC, 12, 20, –10) from a previous study [27], which reported that the pACC is involved in reward-associated decision making, while the sACC is related to the resultant reward of the decision. We then extracted the beta estimates averaged in each of those ROIs using the MarsBaR-toolbox (http://marsbar.sourceforge.net/). We also constructed an additional GLM with three difficulty levels (Hard, Medium, and Easy). To this end, we concatenated the scans from the separate sessions, and divided all trials (apart from those under the Control condition) into three difficulty levels according to the length of RT (long, intermediate, and short, respectively) for each participant. The effects of transition between sessions were taken into account with regressors of sessions. For the ROI-based beta estimate analyses, we used the statistical threshold of uncorrected *P* < 0.05 according to the exploratory purpose of these analyses.

## Results

### Behavioural Results

**Fig 2:**
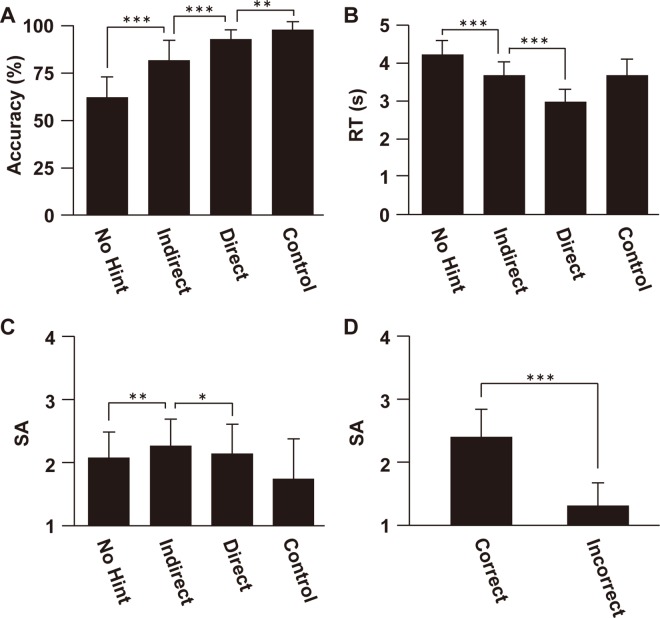
Behavioural data for the four conditions. Accuracy (A), RT (B), and SA (C) are shown for the four conditions (two notations concatenated). (D) SA in the correct and incorrect trials, averaged across No hint, Indirect, and Direct conditions. *P < 0.05. **P < 0.01. ***P < 0.001. Error bars, SD.

Regarding accuracy, two-way repeated measures analysis of variance (rANOVA) showed that the main effect of notation (Number and Letter) [*F*(1, 20) = 15, *P* < 0.001] and the main effect of condition (No hint, Indirect, Direct, and Control) [*F*(3, 60) = 111, *P* < 0.001] were significant (Fig 2A). The interaction was also significant [*F*(3, 60) = 2.8, *P* = 0.048]. Next we performed post-hoc paired *t*-tests among the four conditions on the notation-concatenated data. As a result, we found significant difference of accuracy between the No hint and Indirect conditions (*P* < 0.001), between the Indirect and Direct conditions (*P* < 0.001), and between the Direct and Control conditions (*P* = 0.0016) (in summary, Control > Direct > Indirect > No hint). Similar patterns were observed when we analysed the accuracy in Number and Letter notations separately (S2A and S2B Fig). As for RTs, the main effect of notation [*F*(1, 20) = 11.1, *P* = 0.0034] and the main effect of condition [*F*(3, 60) = 44.3, *P* < 0.001], were significant (Fig 2B). The interaction was also significant [*F*(3, 60) = 5.8, *P* = 0.0015]. Post-hoc paired *t*-tests showed significant difference of RTs between the No hint and Indirect conditions (*P* < 0.001), as well as between the Indirect and Direct conditions (*P* < 0.001) (in summary, No hint > Indirect > Direct). The direct comparisons between the Control and other conditions were not performed because in the Control condition participants were asked to always respond after 4 s of the problem onset. Similar patterns were observed when we analysed the RTs in Number and Letter notations separately (S2C and S2D Fig). The number of trials where RT exceeded 5 s was 9.1±5.1 (average±s.d.) for the No hint condition, 5.7±4.3 for the Indirect condition, and 2.2±2.3 for the Direct condition (the total number of trials was 32 for each condition). Regarding SA, the main effect of notation [*F*(1, 20) = 11.5, *P* = 0.0029], as well as the main effect of condition [*F*(3, 60) = 14.7, *P* < 0.001], was significant (Fig 2C). The interaction was not significant [*F*(3, 60) = 1.7, *P* = 0.18]. To examine the influence of different instruction types on SA, we performed paired *t*-tests among the four conditions on the notation-concatenated data. As a result, we observed that the Indirect condition induced the largest SA (*P* < 0.05). We also found that SA of the Direct, Indirect, and No hint conditions was larger than that of the Control condition (*P* < 0.01). Similar patterns of SA were found regardless of notation difference (S2E and S2F Fig). Next we divided all conditions into correct and incorrect trials (Fig 2D). By comparing SA in correct and incorrect trials, we found that SA in the correct trials was significantly higher than that in the incorrect trials [*t*(21) = 9.0, *P* < 0.001].

**Fig 3:**
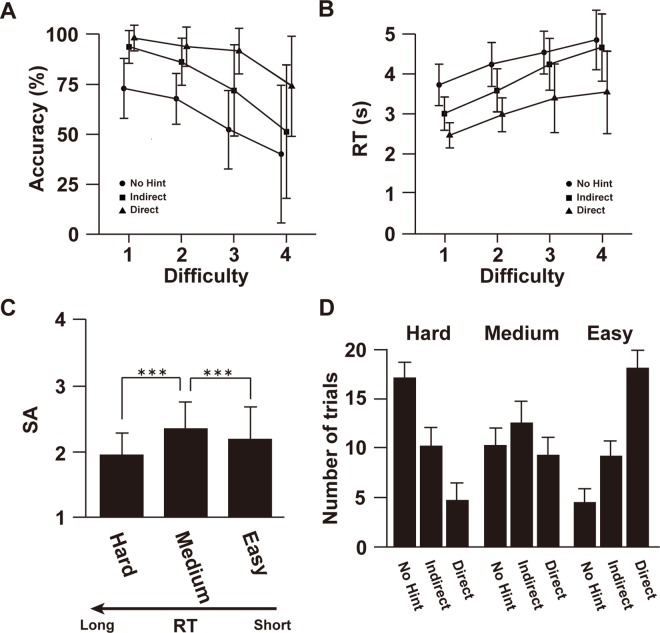
Effect of intrinsic problem difficulty. Accuracy (A) and RT (B) for three instruction levels are shown as a function of intrinsic problem difficulty (Difficulty). (C) SA for the three difficulty levels, divided according to the RT differences for each participant. (D) The average number of trials for each of three instructions included in each difficulty level. ***P < 0.001. Error bars, SD.

We further analysed accuracy and RT as a function of intrinsic problem difficulty obtained outside the MR scanner (Fig 3A and 3B), and found that the three instruction types modulated both accuracy and RT. For the RT, two-way rANOVA showed a significant main effect of intrinsic problem difficulty [abbreviated as Difficulty, *F*(3, 54) = 63.5, *P* < 0.001] and condition [*F*(2, 36) = 150.2, *P* < 0.001], however the interaction was not significant [*F*(6, 105) = 1.8, *P* = 0.12]. Regarding Accuracy, the main effects of intrinsic problem difficulty [*F*(3, 54) = 28.4, *P* < 0.001] and condition [*F*(2, 36) = 67.3, *P* < 0.001] were significant, however the interaction was not significant [*F*(6, 105) = 1.5, *P* = 0.12]. These effects were also observed when we examined both Number and Letter notations separately (S3A–S3D Fig), although the overall intrinsic problem difficulty was different between two notations [*t*(21) = 3.6, *P* < 0.001, S3E Fig]. These results show that, despite the variability in intrinsic problem difficulty, our instruction design successfully produced three different levels regardless of the notation.

To diminish the influence of the intrinsic problem difficulty and individual variability, we divided all trials (apart from those under the Control condition) into three difficulty levels (Hard, Medium, and Easy) according to the length of RTs (see S4 Fig for two representative participants’ data). We again performed paired *t*-tests among three difficulty levels, and found that the Medium level (with intermediate difficulty) had the largest SA (*P* < 0.001, Fig 3C). We also confirmed that the three levels of instructions corresponded well to the three difficulty levels (Fig 3D). Since similar patterns for SA were found when we examined two notations separately (S5 Fig), we concatenated Number and Letter notations in the following fMRI analysis.

### fMRI Results

**Fig 4:**
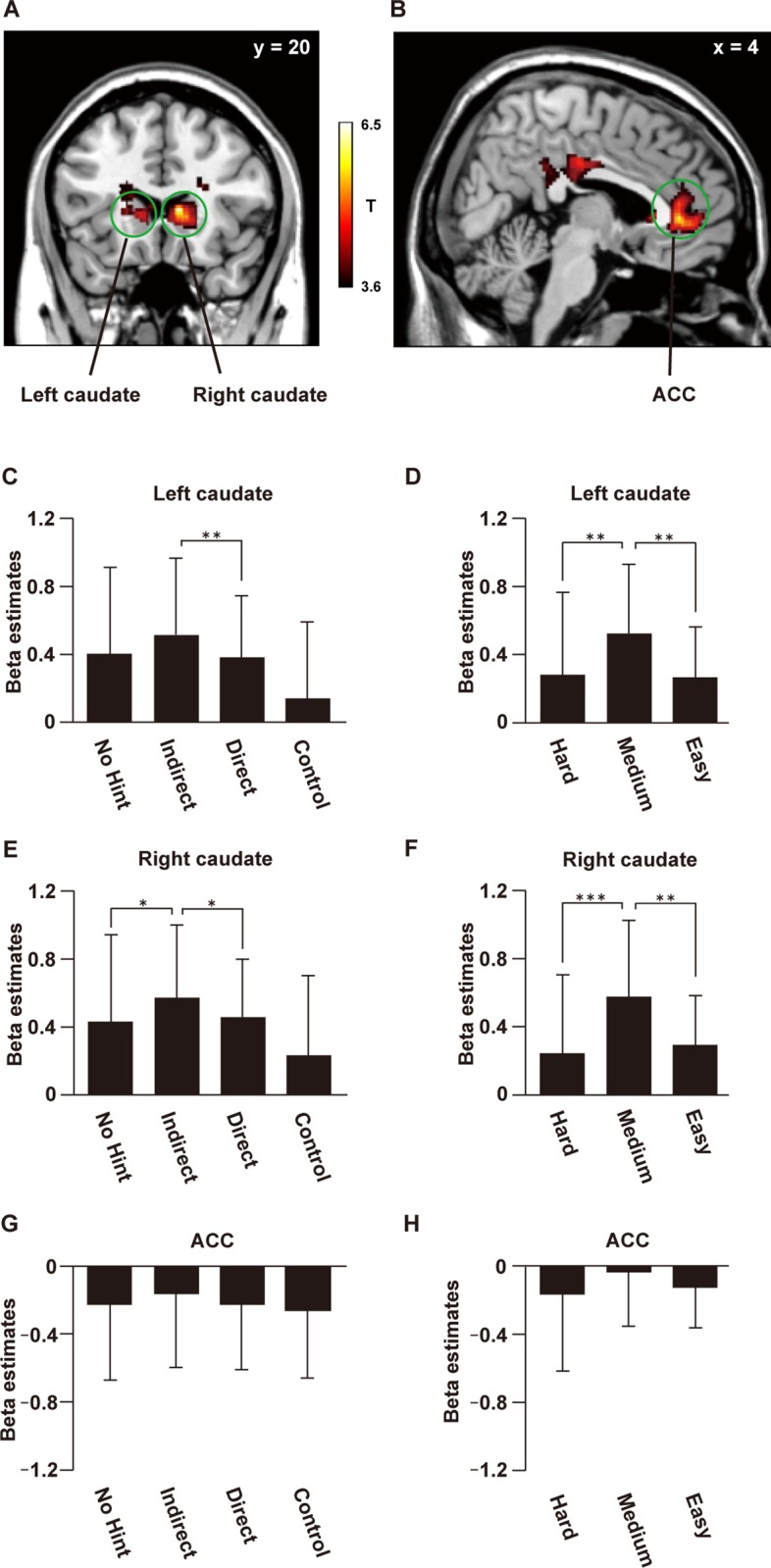
Neural activations parametrically modulated by SA. The cortical activation map was projected onto coronal (A) and sagittal (B) plains (*P* < 0.001 for voxel level, *P* < 0.05 for cluster level, with topological FDR correction). See Table 1 for stereotactic coordinates. Beta estimates for four conditions were extracted from anatomical ROIs of the left caudate (C), the right caudate (E), and ACC (G). (D, F, G) In the same ROIs, we extracted beta estimates for the three difficulty levels divided according to the length of RT. *P < 0.05. **P < 0.01. ***P < 0.001. Error bars, SD.

To identify the cortical regions reflecting notation-independent SA during the problem-solving period, we performed a one-sample *t*-test with images of positive modulation by SA for each participant (Fig 4A and 4B, Table 1). We found significant modulation including the bilateral caudate, ACC [Brodmann’s areas (BA) 11], and posterior cingulate cortex (PCC, BA 23). We also found modulation in the left ventrolateral prefrontal cortex (vlPFC, BA 47), left ventromedial prefrontal cortex (vmPFC, BA 11), right middle occipital gyrus (MOG, BA 18), and right lateral occipital gyrus (LOG, BA 19). No region showed significant negative modulation with SA. When we performed the whole brain analysis in each instruction level (uncorrected *P* < 0.001, S7 Fig), we found significant positive modulation with SA in the ACC, PCC, and ventral putamen under the No Hint condition, modulation in the ACC under the Indirect condition, and modulation in the bilateral caudate under the Direct condition. None of those regions were modulated under the Control condition.

**Table 1:** Regions where activations were modulated by SA.

Brain region	BA	Side	*x*	*y*	*z*	*Z*-value	Voxels
vmPFC	11	L	–18	42	–6	4.2	503
			–20	50	–2	3.8	*
vlPFC	47	L	–34	44	–6	4.1	*
ACC	11	M	4	38	2	4.4	1854
Caudate		L	–20	24	4	4.7	*
		R	10	20	6	4.6	*
		R	18	–8	22	3.8	414
			26	–24	24	3.7	*
Thalamus		R	18	–16	16	3.6	*
PCC	23	M	–2	–20	34	4.7	622
			–6	–6	28	4.1	*
MOG	18	R	16	–98	0	3.9	867
			22	–82	–6	3.7	*
LOG	19	R	36	–86	–6	3.6	*

Stereotactic coordinates (*x*, *y*, *z*) in the MNI space (mm) are shown for each activation peak of *Z*-values. ACC, anterior cingulate cortex; PCC, posterior cingulate cortex; vmPFC, ventromedial prefrontal cortex; vlPFC, ventrolateral prefrontal cortex; MOG, middle occipital gyrus; LOG, lateral occipital gyrus; BA, Brodmann’s area; L, left hemisphere; R, right hemisphere; M, medial.

*The region with an asterisk is included within the same cluster shown one row above.

According to our a priori interest in the reward and emotion-related systems, next we examined beta estimates in the anatomical ROIs of the bilateral caudate and ACC (Fig 4C, 4E and 4G, S8 and S9 Figs). To confirm that the activation pattern is independent of notations, we used two-way rANOVA. As a result, we found the significant main effect of condition in the bilateral caudate [left caudate: *F*(3, 60) = 6.2, *P* < 0.001, right caudate: *F*(3, 60) = 5.0, *P* = 0.0037]. In these regions, the main effect of notation [left caudate: *F*(1, 20) = 1.9, *P* = 0.18, right caudate: *F*(1, 20) = 1.2, *P* = 0.29], as well as the interaction [left caudate: *F*(3, 60) = 0.77, *P* = 0.52, right caudate: *F*(3, 60) = 0.42, *P* = 0.73], was not significant. In the ACC, we did not find the significant main effect of condition [*F*(3,60) = 0.34, *P* = 80], the main effect of notation [*F*(3,60) = 0.0080, *P* = 0.93], or the interaction [*F*(3,60) = 0.55, *P* = 0.65]. Indeed, paired *t*-tests for the notation-concatenated data revealed that the Indirect condition induced the largest activations in the right caudate among the three instruction levels (*P* < 0.05). In the left caudate, a significant difference was found only between the Indirect and Direct conditions (*P* = 0.0070).

**Fig 5:**
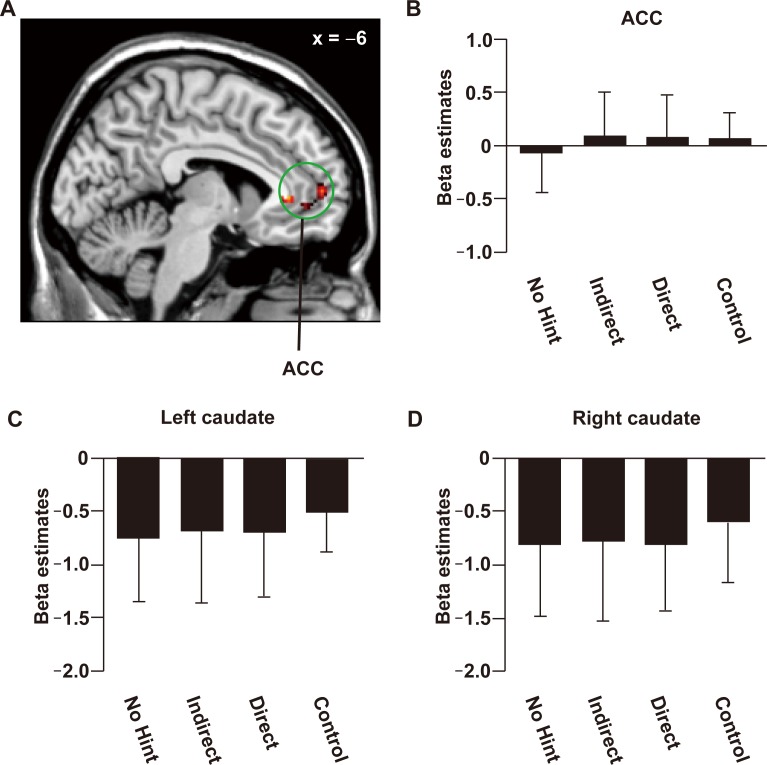
Activation modulated by SA in the answer-feedback period. The cortical activation map was projected onto the sagittal plain (uncorrected *P* < 0.001). See Table B in S1 File for the stereotactic coordinates.

As above, we divided all trials (apart from those under the Control condition) into three difficulty levels (Hard, Medium, and Easy) according to the length of RTs (Fig 4D, 4F and 4H), since we confirmed through the behavioural data that this division should be sensitive to the individual variability and effect of intrinsic problem difficulty. This analysis revealed that Medium level trials showed significantly larger activations than other levels in the bilateral caudate (*P* < 0.01), consistent with our behavioural data. Activations in the ACC were negative (Fig 4G and 4H, S9E and S9F Fig), and we did not find the significant main effect of condition [*F*(3, 60) = 0.34, *P* = 0.80], or the main effect of notation [*F*(1, 20) = 0.008, *P* = 0.93]. The interaction was not significant [*F*(3, 60) = 0.55, *P* = 0.65]. Even after dividing all trials into three difficulty levels, no significant difference in activation was found among those levels in the ACC [*F*(2, 40) = 1.7, *P* = 0.20, Fig 4H), suggesting different roles for the bilateral caudate and ACC concerning SA. The difference between the caudate and ACC was further indicated by the analysis of the answer-feedback period, which showed activations modulated by SA in the ACC, but not in the caudate (uncorrected *P* < 0.001, Fig 5 and Table B in S1 File).

**Fig 6:**
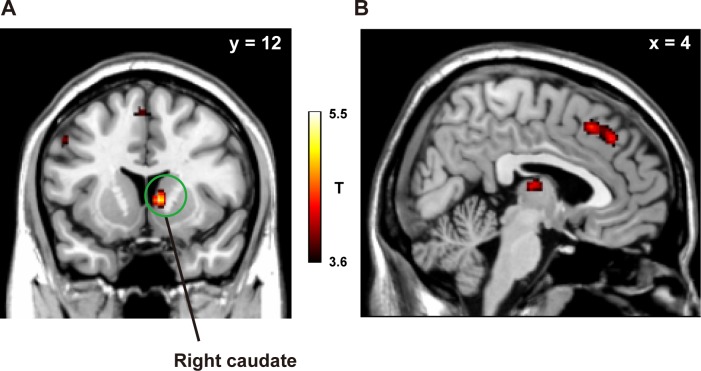
Activation modulated by SA only for the correct trials. Neural activations parametrically modulated by SA, only for the correct trials. The cortical activation map was projected onto the coronal (A) and sagittal (B) plains (uncorrected *P* < 0.001). See Table C in S1 File for the stereotactic coordinates.

**Fig 7:**
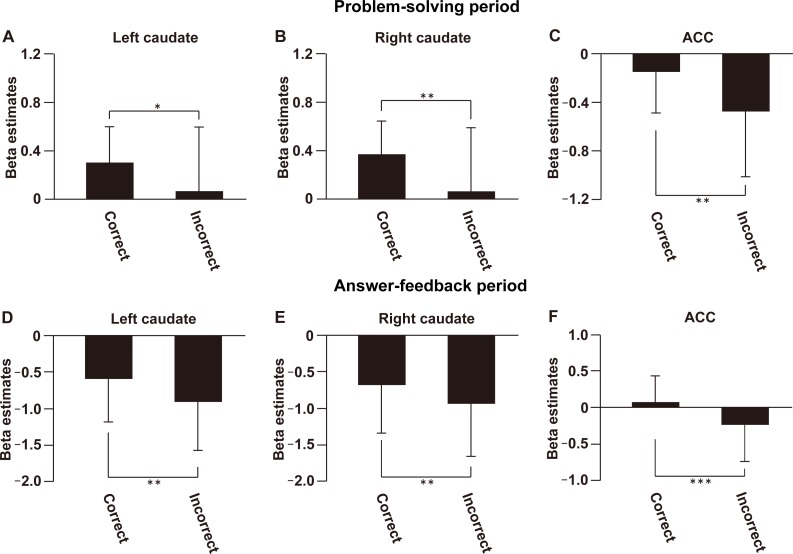
Activations in the correct and incorrect trials. Beta estimates of correct and incorrect trials were extracted from the anatomically defined ROIs (S7 Fig) of the left caudate (A, D), right caudate (B, E), and ACC (C, F), for both problem-solving period and answer-feedback period. *P < 0.05. **P < 0.01. ***P < 0.001. Error bars, SD.

To show that our results of SA modulation is robust regarding the trial selections and effect of accuracy, we performed an additional parametric modulation analysis only for the correct trials (Fig 6). We found significant modulation in the right caudate, but not in the ACC (uncorrected *P* < 0.001). Next we analysed activations in the correct and incorrect trials separately (without the Control condition). In the problem-solving period, in each of the three regions we found that correct trials induced larger activations than incorrect trials [left caudate: *t*(21) = 2.5, *P* = 0.011, right caudate: *t*(21) = 2.9, *P* = 0.004, ACC: *t*(21) = 3.4, *P* = 0.001, Fig 7A–7C]. In the answer-feedback period, in each of the three regions we again found that correct trials induced larger activation than incorrect trials [left caudate: *t*(21) = 3.2, *P* = 0.002, right caudate: *t*(21) = 2.7, *P* = 0.007, ACC: *t*(21) = 3.5, *P* < 0.001, Fig 7D–7F]. Those results may indicate that activations in the ACC basically reflect the differences between correct and incorrect trials.

## Discussion

In the present study, we prepared three levels of instruction and a basic control for the same insight-problem types, and we obtained following results. First, we found that the Indirect hint induced the largest SA among the three instruction levels. We also confirmed that the Indirect hint corresponded well to the intermediate level of difficulty for each participant. Secondly, using fMRI we observed that activations in the bilateral caudate, together with the ACC, PCC, vmPFC, and vlPFC, showed significant modulation with SA during the problem-solving period. Thirdly, in the bilateral caudate, the Indirect hint induced the largest activations. This effect was also found when we focused on the intermediate difficulty trials for each participant. Fourthly, during answer-feedback period, the activation in the ACC was modulated by SA, while the ACC activation seemed to reflect differences between the correct and incorrect trials. Lastly, we found that such activation patterns were notation-independent. These results indicate that the Indirect instruction can most effectively contribute to the learners’ SA, and that such feeling is principally processing by the bilateral caudate in the reward system of the brain.

Controversy surrounds the education science community concerning the efficiency of minimal instruction versus direct instruction [1]. Our current results indicate that both extremes are ineffective, and that the Indirect hint has a positive influence on the participants’ SA. Although the participant obtained highly accurate performance with direct instructions (Fig 2 and S2 Fig), it seemed that they did not report satisfaction because the tasks had become too easy. Similarly, the No hint condition also induced a reduced SA in comparison to the Indirect condition, mirroring the lower success rate under this condition. Under the Indirect condition, the participants were given partial information regarding problem solving. Such instruction allows the participant to discover information by himself or herself, considered an example of “guided discovery” [1, 6].

Research shows that tasks with intermediate difficulty induce the greatest amount of pleasure after solving the task [7–8]. Our results are consistent with this view, since the Indirect hint successfully imposed an intermediate difficulty level, and induced the largest SA among the three instruction levels (Fig 2A–2C). Furthermore, another previous study also indicated that the strength of motivation to perform a task was maximised with intermediate difficulty and with the largest uncertainty of success [28]. Our results provide new evidence to bridge the gap between task difficulty, SA, and motivation for learning.

The bilateral caudate has been reportedly involved in reward-based learning [16], as well as in motivation studies [13–14]. Other imaging study also suggests involvement of caudate in the artificial grammar learning of letter sequences [29]. Although the authors did not discuss the involvement of caudate in the reward-processing, caudate activation in the contrast of grammatical vs. nongrammatical sequences may reflect SA with successful grammatical judgments. In the current experiment, we found that the bilateral caudate principally reflected SA during problem-solving period, which was mirroring behavioural results of the superiority of the Indirect hint, supporting our assumption that the caudate is involved in motivation-enhancing aspect in learning. When we analysed SA modulation in each instruction level, we also found modulation in the ventral putamen under the No hint condition (S7A Fig), but the subsequent ROI analysis indicated that activations in the ventral putamen were very weak or negative even during problem-solving period (S11 Fig). Although the left NAcc showed some tendency of SA-like activation patterns, the difference between No hint and Indirect conditions were not as clear as in the caudate ROIs (S12 Fig). It has been reported that dorsal striatum (including caudate) is involved in the maintenance of information about the rewarding outcomes necessary for learning, while ventral striatum is simply involved in the anticipation of future reward [18]. Our results suggest that the sense of accomplishment is processed rather in the caudate which has particular importance in motivation for learning.

Studies have shown that the activation of the ACC is related to the processing of emotional saliency [20–21]. Our experimental results indicate that the reward system interacts with this emotion processing system. Furthermore, activation in the PCC was observed for the processing of emotional words [30], and emotion-based moral judgments [31]. As for the negative activations in the ACC during problem-solving period, it is possible that this negative BOLD is due to the task-specific deactivation of the default mode network [32]. However, considering the modulation by SA, such baseline deactivation may be further affected by emotional saliency. During the answer-feedback period, we found modulation with SA in the ACC/vmPFC, but not in the bilateral caudate (Fig 5 and Table B in S1 File), which is consistent with a previous neuroimaging study which reported activation in the ACC/vmPFC for reward outcomes [33]. The fROI analysis showed that activations in the ACC were the largest under the indirect instructions during the answer-feedback period (S10F Fig). The separate analyses of ACC subparts indicate that such SA-like activation patterns derive from the pACC, but not from the sACC (S13 Fig). A previous imaging study reported that pACC is more involved in reward-associated decision making compared to the sACC [27], which is relevant to the problem solving activity in the present experiment. However, we did not find modulation of activations in the ACC when we analysed only with the correct trials (Fig 6). It is conceivable that the ACC activation may be induced by conflict resolution (i.e., knowing the correct answer to the given problems), in such case the incorrect trials are with “higher conflict state” and may induce larger activation. However, activations in the ACC were larger in the correct trials than in the incorrect trials (Fig 7C and 7F), even during the answer-feedback period. This result cannot be explained by the conflict-resolution interpretation, suggesting that the ACC likely reflects the differential emotional saliency between the correct and incorrect trials rather than SA itself.

Many fMRI studies have observed an increase in activations, specifically in the frontal and parietal regions, in tasks with high cognitive demands compared to those with low demands, across various cognitive domains [34–35]. Indeed, we found activation in the left IFG, left LPMC, and bilateral parietal cortex when the participants solved problems compared to the Control condition (S14 Fig). Among those activated regions, left-lateralized activation in the IFG and LPMC may reflect recursive computation of sequential symbols common to language and mathematics [36]. However, we also showed that higher task difficulty does not always induce larger activations in reward systems. This view is supported by a recent neuroimaging study, which revealed that both cognitive effort and motor effort modulated activations in the bilateral striatum, independent of task difficulty [37]. Although two notations (Number and Letter) had different intrinsic problem difficulties (S3E Fig), the effect of the Indirect condition was robust. Our results show that the reward and emotion-related regions contribute to SA in a domain-general manner, most likely playing a complementary role with front-parietal networks during problem solving.

By dividing all trials into groups according to the length of the RT, we considered individual variability in their skills. We found that trials with intermediate RTs induced the largest SA (Fig 3C and 3D and S5 Fig), and the largest activations in the bilateral caudate (Fig 4D and 4F). Since indirect instructions correspond well to the trials with intermediate RTs, indirect instructions may be effective regardless of individual skill. It is possible that some individuals prefer to solve problems by themselves, whereas others prefer to be instructed step by step. Therefore, it remains important to adjust problem difficulty and instruction type to each individual to obtain an optimal efficiency for problem solving. Moreover, based on the expectancy-value theory, the effect of problem difficulty on the attitude toward success may depend on the personality trait of motivation to achieve success and avoidance of failure [38]. A future study may clarify the relationship between individual variability of motivation toward learning and SA/activation in the reward system.

It would be important to discuss some technical limitations in the current experimental setting. First, although in the problem-solving period the trial onsets were modeled based on the participants’ response timing and were automatically jittered in each trial (with a low correlation coefficient value of regressors shown in Supporting Information), in the answer-feedback period the trial onsets were fixed. This experimental setting may lead to the underestimation of the effects in the answer-feedback period. Secondly, for the sake of fMRI analysis based on the participants’ button presses, we instructed participants to press any button when they couldn’t find an answer. Under this experimental design we could not distinguish between participants’ random guess and the true insight. However, those forced responses would result in the slow RT and thus included in Hard trials, and in such unsuccessful trials SA would be small. In the current paradigm the Medium trials are the most important component for considering the effect of SA on the caudate. Therefore the negative effect of this instruction was limited.

The application of neuroscience to education, called “Neuroeducation”, has recently attracted a wide variety of attention [11–12]. Our current results may show a striking impact on this field, by giving a neuroscientific basis on instructional methods for students. We conclude that reported sense of accomplishment is effectively induced by the Indirect instruction method, and represented in the brain’s reward system. Our present results are an important source for considering the controversy on the efficacy of instructions [1], and will provide new insights on the sense of accomplishment and brain reward system.

## Supporting Information

S1 Fig Examples of original stimuli. Original Japanese stimuli are shown in Number (A) and (B) Letter notations.
(TIF)

S2 Fig Accuracy, RT, and SA for both notations. Accuracy (A) (B), RT (C) (D), and SA (E) (F) are shown for the four conditions, in both Number and Letter notations. *P < 0.05. **P < 0.01. Error bars, SD.
(TIF)

S3 Fig Effect of intrinsic difficulty for both notations. Accuracy (A) (C) and RT (B) (D) in three levels of instructions are shown as a function of intrinsic problem difficulty (Difficulty), analysed separately for Number and Letter notations. (E) Difference of Difficulty (averaged for all conditions) between two notations. ***P < 0.001. Error bars, SD.
(TIF)

S4 Fig Examples of the RT distribution categorized in three difficulty levels. The distributions of RTs were shown for two representative participants (A, B). The number of trials for each difficulty level (Easy, Medium, Hard) was described in a stacked histogram.
(TIF)

S5 Fig Three difficulty level analysis shown for both notations. (A) (B) SA for the three difficulty levels, divided according to the RT difference, and (C) (D) the average number of trials for three instructions included in each difficulty level, analysed separately for Number and Letter notations. **P < 0.01. ***P < 0.001. Error bars, SD.
(TIF)

S6 Fig Transfer effect among conditions. In the 1st session, RT differences between initially-appeared problems and secondary-appeared problems were calculated for the No hint to Indirect, Indirect to No hint, Direct to Control, and Control to Direct directions. *P < 0.05. Error bars, SD.
(TIF)

S7 Fig Positive modulation with SA in each instruction level. The cortical activation map was projected onto the coronal (A) and sagittal (B) plains for the No hint condition, coronal plain for the Direct condition (C), and sagittal plain for the Indirect condition (D) (uncorrected *P* < 0.001).
(TIF)

S8 Fig Anatomical ROIs of the Automatic Anatomical Labeling atlas. Anatomically defined ROIs of the left caudate (A), right caudate (B), and ACC (C) were projected onto the coronal (A-B) and sagittal (C) plains.
(TIF)

S9 Fig Beta estimates for both notations. Beta estimates were extracted from the anatomical ROIs of the left caudate (A, B), right caudate (C, D), and ACC (E, F), analysed separately for Number and Letter notations. Error bars, SD.
(TIF)

S10 Fig Beta estimates in the functionally defined ROIs. Beta estimates were extracted from the functionally defined ROIs of the left caudate (A, D), right caudate (B, E), and ACC (C, F) for both problem-solving period and answer-feedback period. *P < 0.05. Error bars, SD.
(TIF)

S11 Fig Anatomical ROI analysis in the ventral putamen. Beta estimates were extracted from the anatomically defined ROIs of the bilateral ventral putamen determined based on the previous study [18].
(TIF)

S12 Fig Anatomical ROI analysis in the nucleus accumbens. Beta estimates were extracted from the anatomically defined ROIs of the bilateral nucleus accumbens (NAcc) determined based on the previous study [19].
(TIF)

S13 Fig Anatomical ROI analysis in the ACC subparts. Beta estimates were extracted from the anatomically defined ROIs of the subcallosal ACC (sACC) and pregenual ACC (pACC) determined based on the previous study [27].
(TIF)

S14 Fig Neural activations for each instruction without a parametric modulation regressor by SA. The cortical activation maps of No hint–Control (A) and Indirect–Control (B) were projected onto the standard brain (*P* < 0.001 for voxel level, *P* < 0.05 for cluster level, with topological FDR correction). See Table D in S1 File for stereotactic coordinates. Beta estimates for four conditions were extracted from functionally defined ROIs (left IFG and LPMC) of Indirect–Control contrast (C-F). Error bars, SD.
(TIF)

S1 File Additional analyses and tables. A supporting information file contains the correlation analyses between regressors, the effect of no response trials, analyses of rule-transfer effect, functionally defined region of interest (ROI) analyses, anatomical ROI analyses in the bilateral ventral putamen, anatomical ROI analyses in the bilateral nucleus accumbens (NAcc), anatomical ROI analyses in the ACC subparts, activations without a parametric modulation regressor by SA, and Table A-D.
**Table A in S1 File**. **The number of no response trials.** The average number of no response trials (mean±SD) are shown for each condition, under each notation.
**Table B in S1 File. Regions where activations were modulated by SA in the answer-feedback period**. Stereotactic coordinates (*x*, *y*, *z*) in the MNI space (mm) are shown for each activation peak of *Z*-values. dlPFC, dorsolateral prefrontal cortex; PCG, postcentral gyrus.
**Table C in S1 File. Regions where activations were modulated by SA for the correct trials**. Stereotactic coordinates (*x*, *y*, *z*) in the MNI space (mm) are shown for each activation peak of *Z*-values. SMA, supplementary motor area; dmPFC, dorsomedial prefrontal cortex; PG, parahippocampal gyrus.
**Table D in S1 File**. **Direct comparison among instruction levels.** Stereotactic coordinates (*x*, *y*, *z*) in the MNI space (mm) are shown for each activation peak of *Z*-values. IPL, inferior parietal lobule; OP, occipital pole; IFG, inferior frontal gyrus; LPMC, lateral premotor cortex; OTG, occipitotemporal gyrus. The region with an asterisk is included within the same cluster shown one row above.
(DOCX)
