# Supplementary figures and images for: Sense of Accomplishment Is Modulated by a Proper Level of Instruction and Represented in the Brain Reward System
Source: PLoS One. 2017 Jan 4;12(1):e0168661. doi: 10.1371/journal.pone.0168661 (PMC5215289; doi:10.1371/journal.pone.0168661)

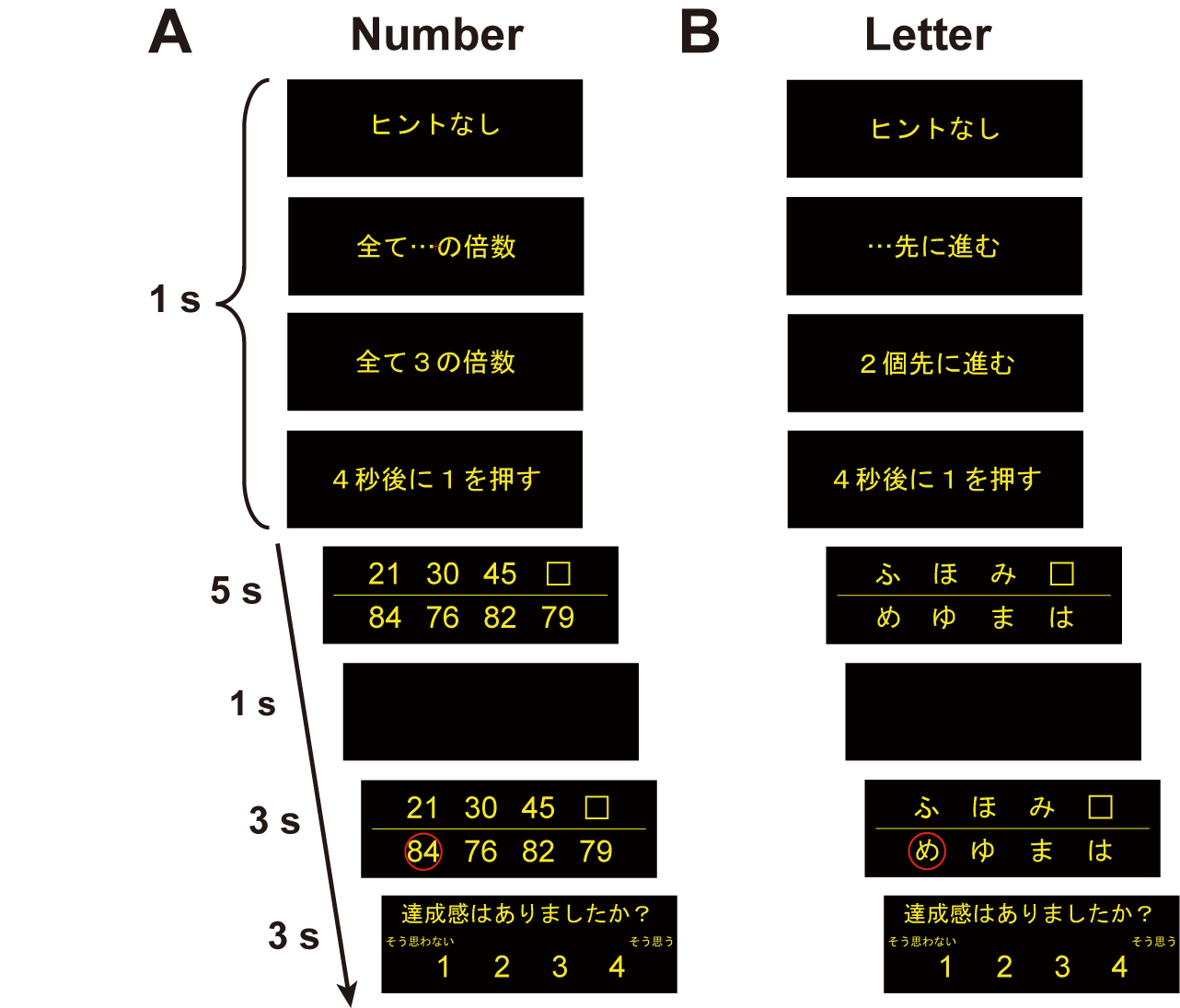

Supplement: S1 Fig — Original Japanese stimuli are shown in Number (A) and (B) Letter notations. (TIF) [file pone.0168661.s001.tif]

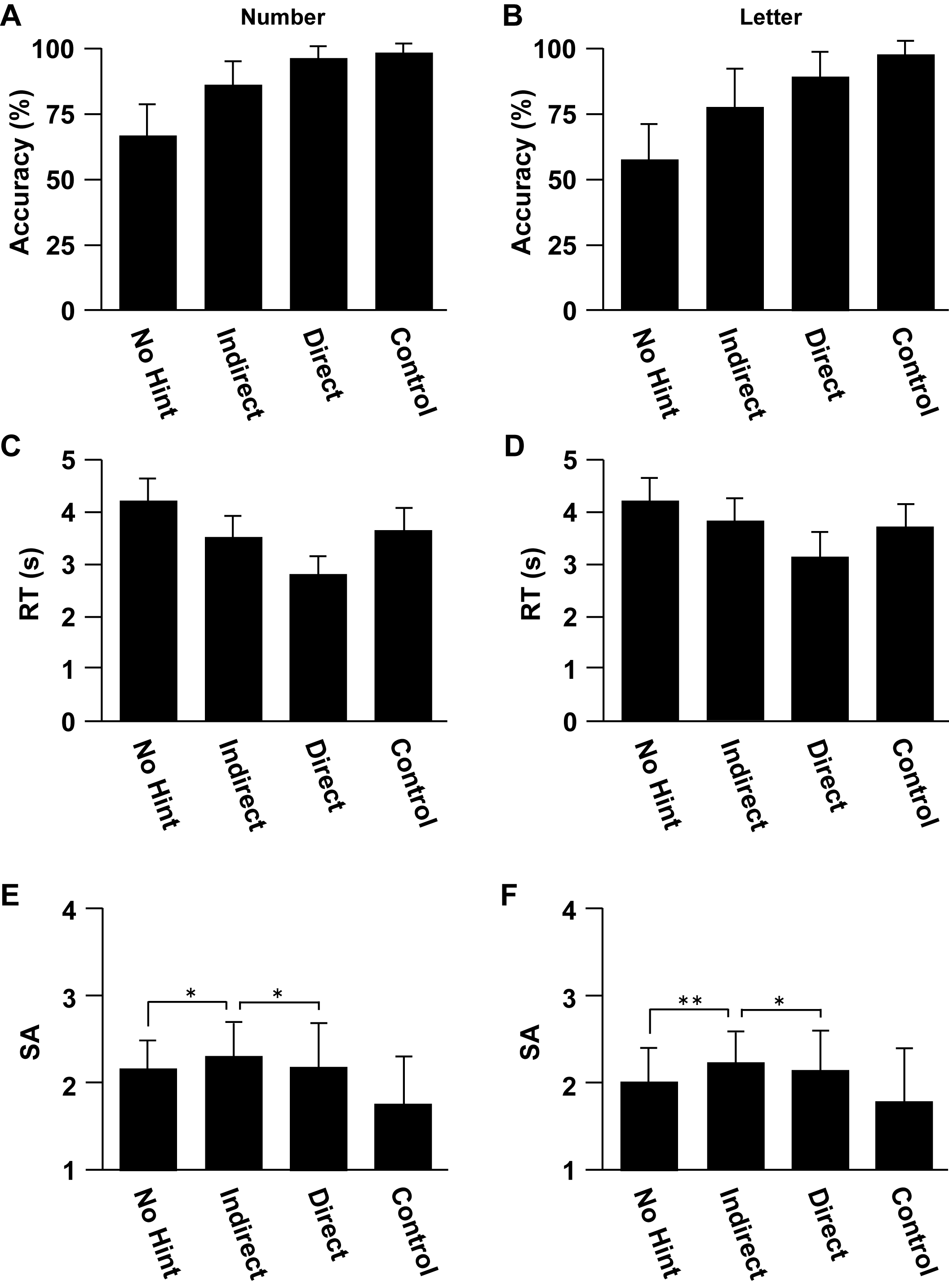

Supplement: S2 Fig — Accuracy (A) (B), RT (C) (D), and SA (E) (F) are shown for the four conditions, in both Number and Letter notations. *P < 0.05. **P < 0.01. Error bars, SD. (TIF) [file pone.0168661.s002.tif]

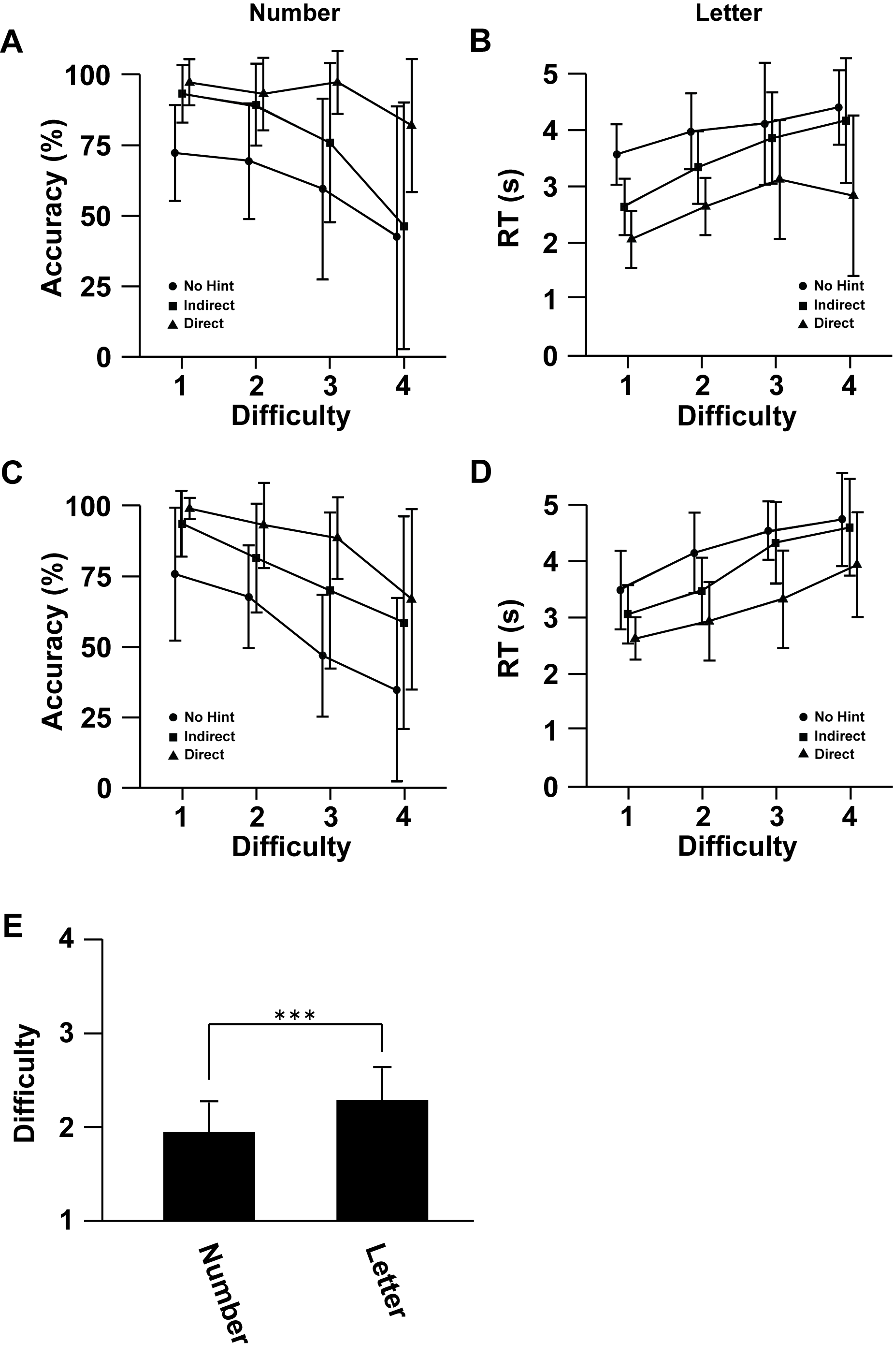

Supplement: S3 Fig — Accuracy (A) (C) and RT (B) (D) in three levels of instructions are shown as a function of intrinsic problem difficulty (Difficulty), analysed separately for Number and Letter notations. (E) Difference of Difficulty (averaged for all conditions) between two notations. ***P < 0.001. Error bars, SD. (TIF) [file pone.0168661.s003.tif]

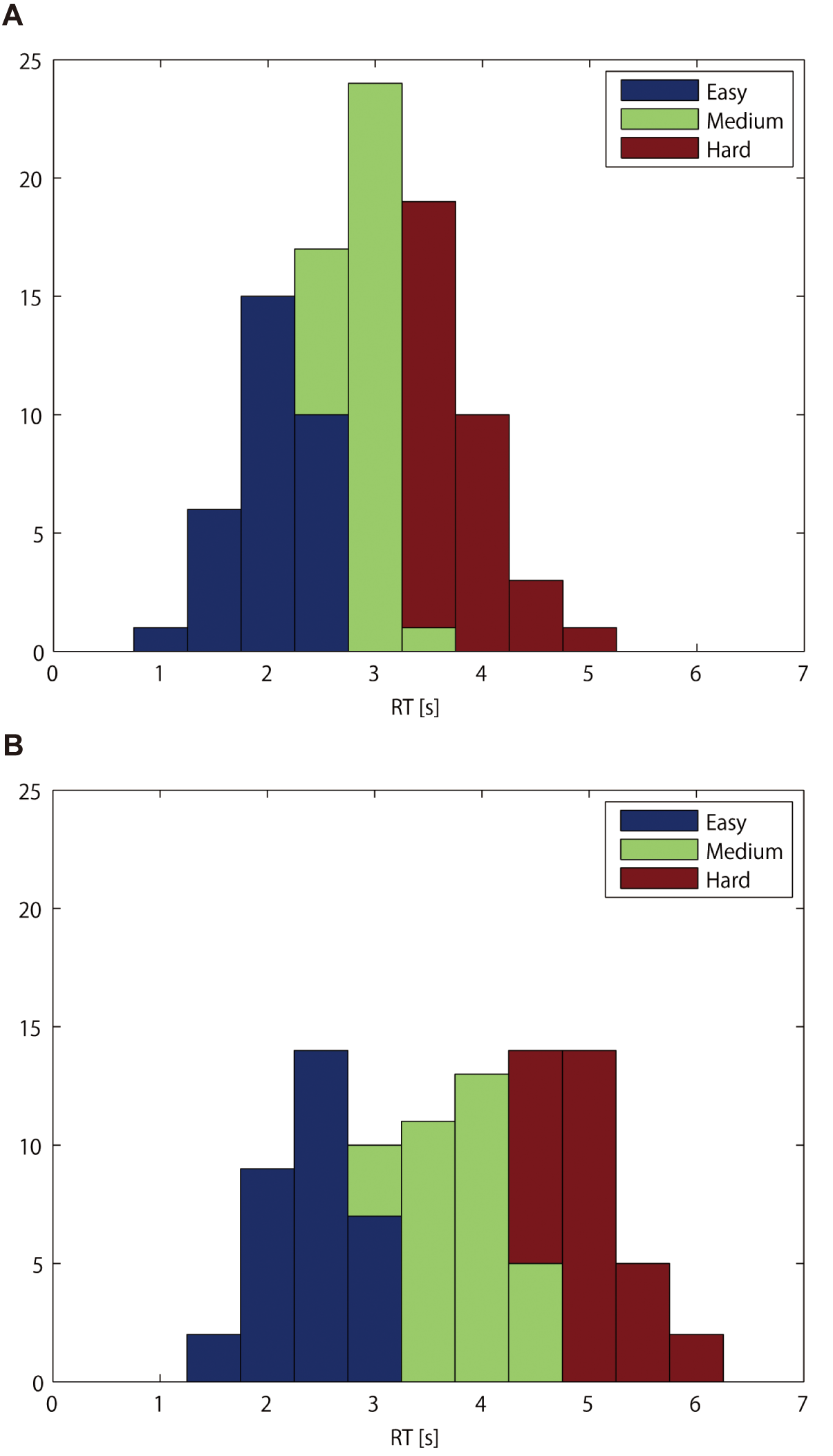

Supplement: S4 Fig — The distributions of RTs were shown for two representative participants (A, B). The number of trials for each difficulty level (Easy, Medium, Hard) was described in a stacked histogram. (TIF) [file pone.0168661.s004.tif]

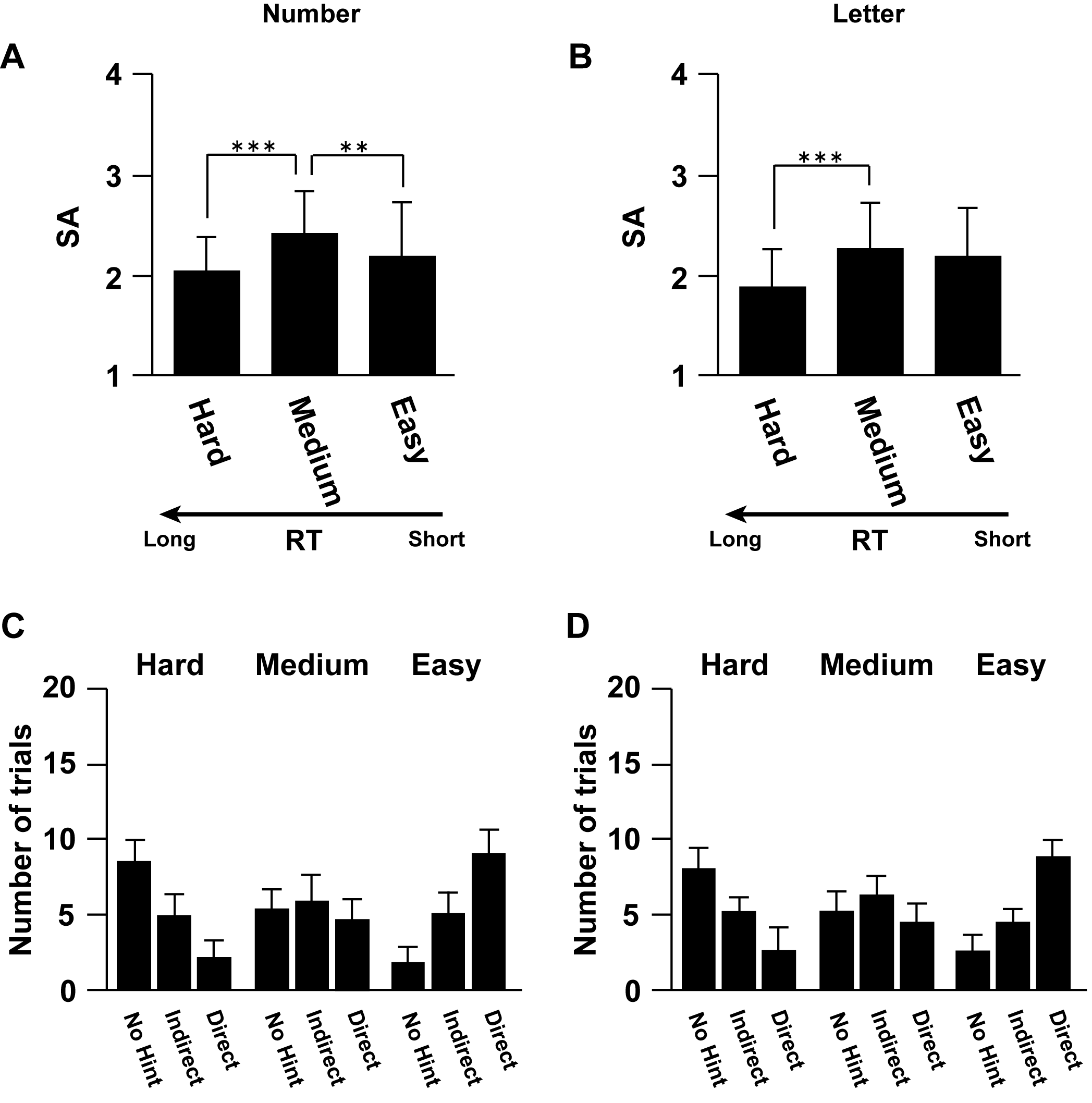

Supplement: S5 Fig — (A) (B) SA for the three difficulty levels, divided according to the RT difference, and (C) (D) the average number of trials for three instructions included in each difficulty level, analysed separately for Number and Letter notations. **P < 0.01. ***P < 0.001. Error bars, SD. (TIF) [file pone.0168661.s005.tif]

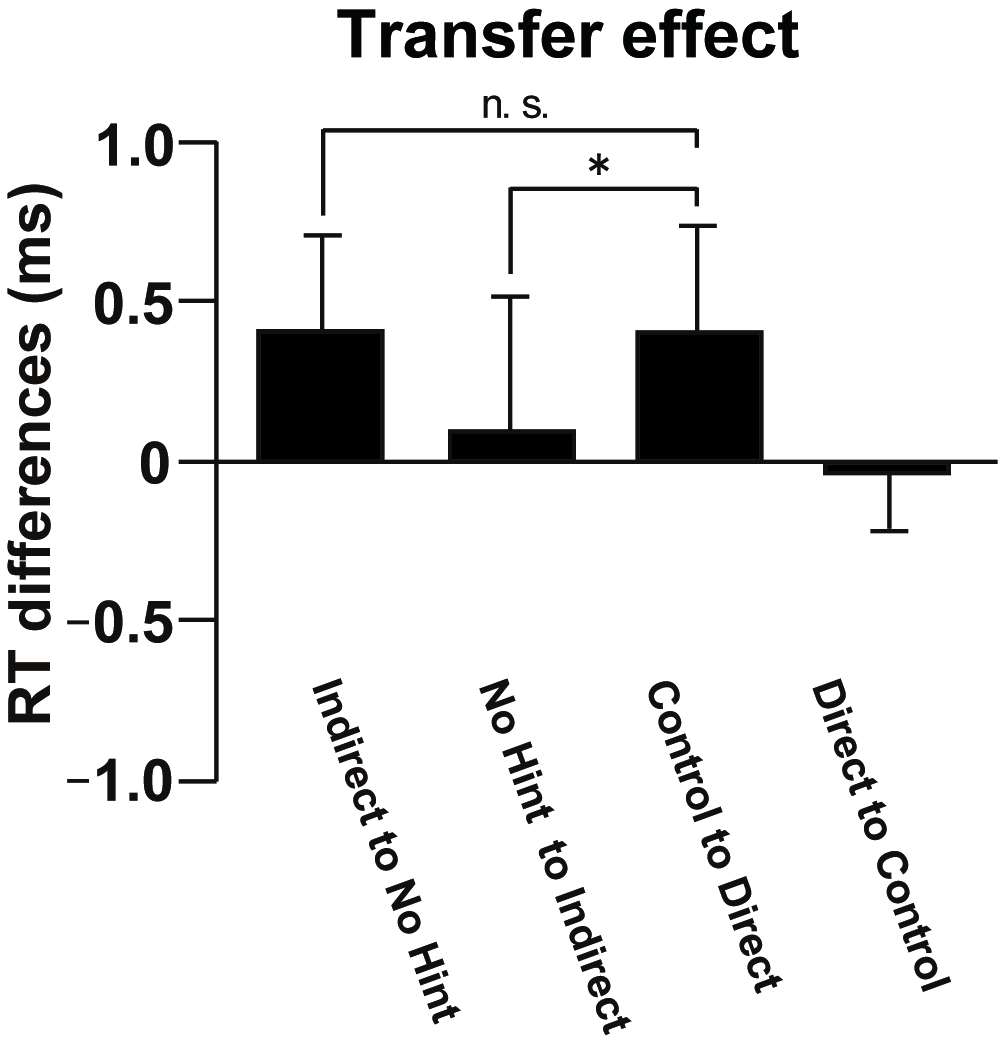

Supplement: S6 Fig — In the 1st session, RT differences between initially-appeared problems and secondary-appeared problems were calculated for the No hint to Indirect, Indirect to No hint, Direct to Control, and Control to Direct directions. *P < 0.05. Error bars, SD. (TIF) [file pone.0168661.s006.tif]

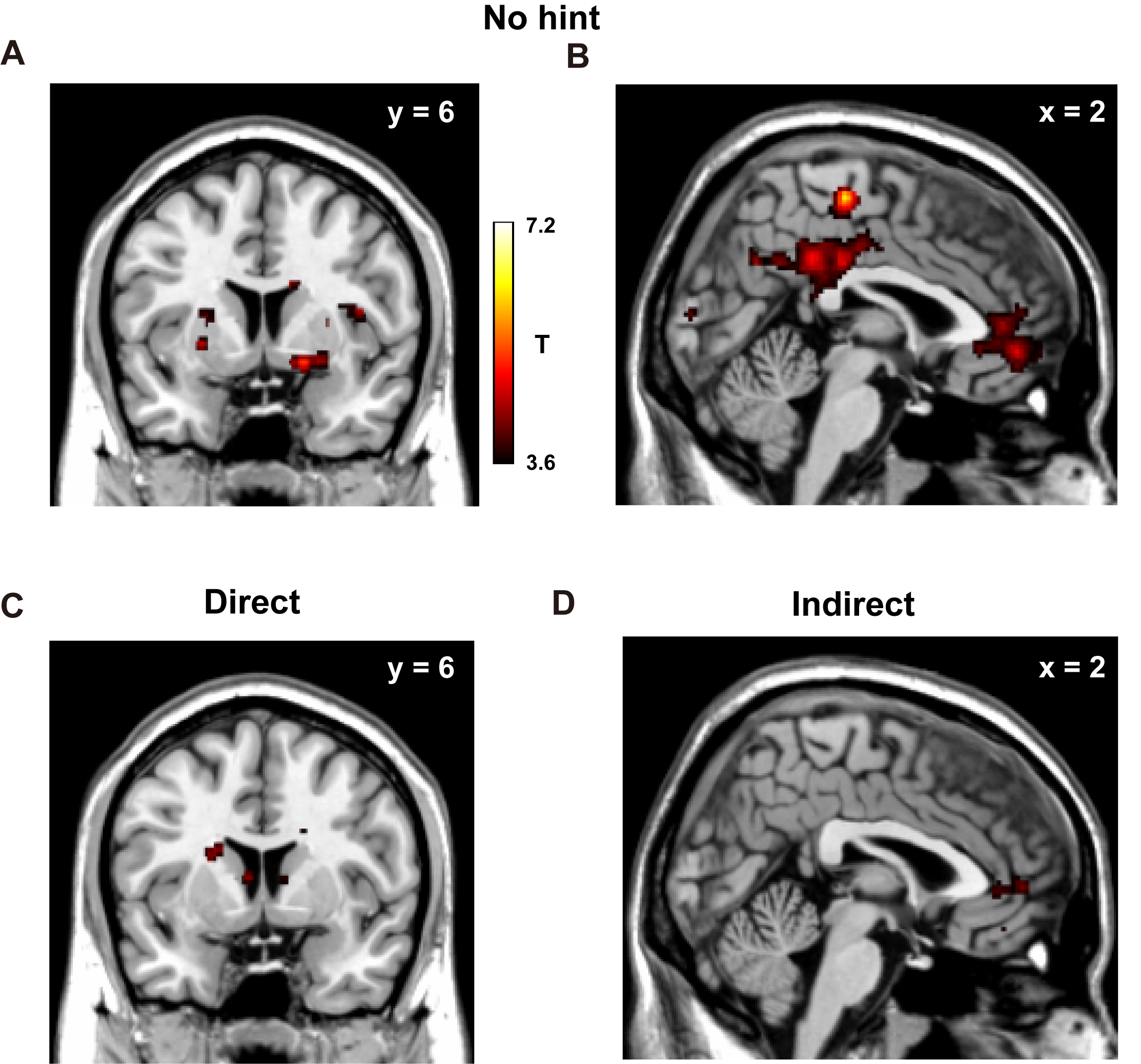

Supplement: S7 Fig — The cortical activation map was projected onto the coronal (A) and sagittal (B) plains for the No hint condition, coronal plain for the Direct condition (C), and sagittal plain for the Indirect condition (D) (uncorrected P < 0.001). (TIF) [file pone.0168661.s007.tif]

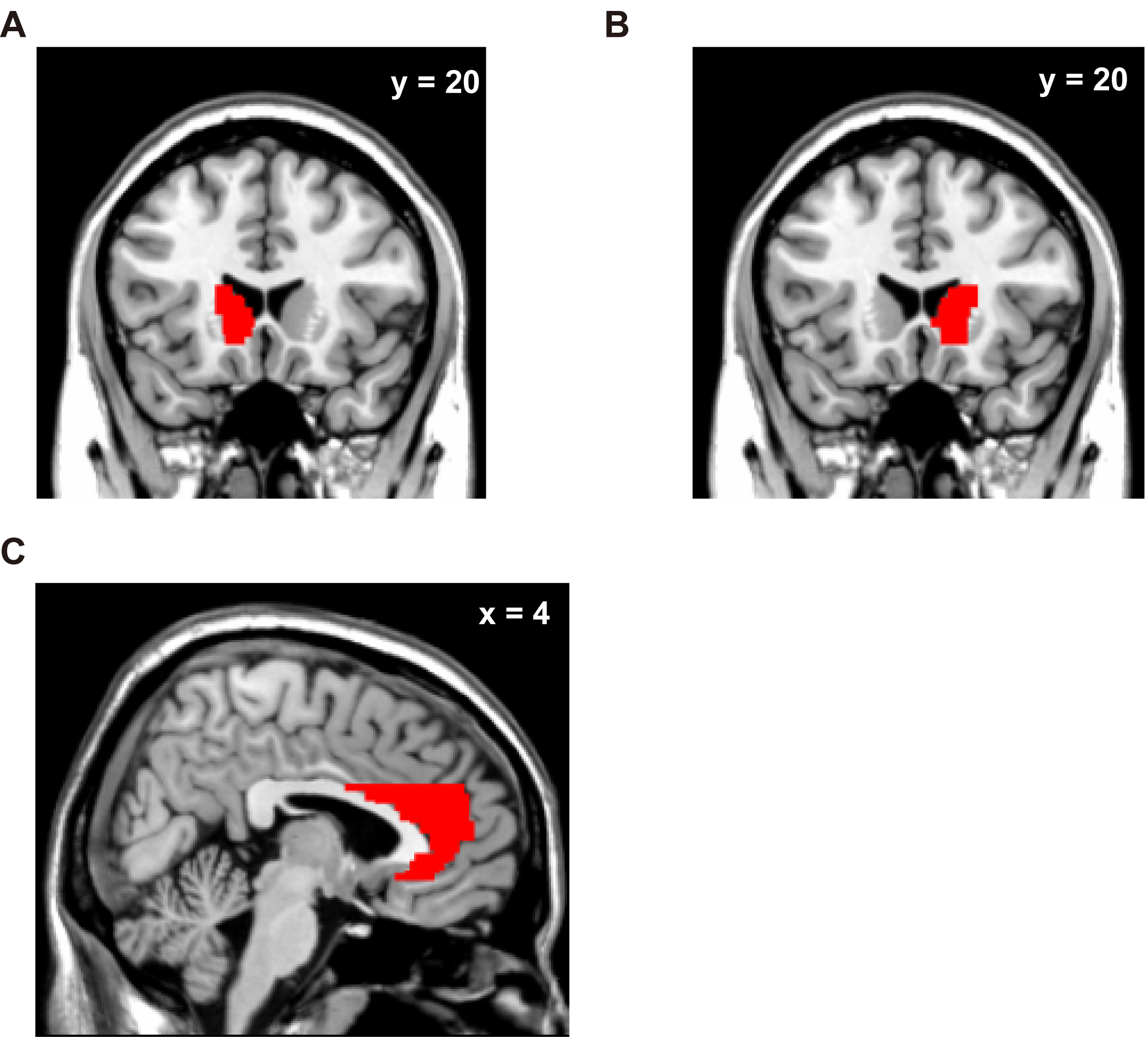

Supplement: S8 Fig — Anatomically defined ROIs of the left caudate (A), right caudate (B), and ACC (C) were projected onto the coronal (A-B) and sagittal (C) plains. (TIF) [file pone.0168661.s008.tif]

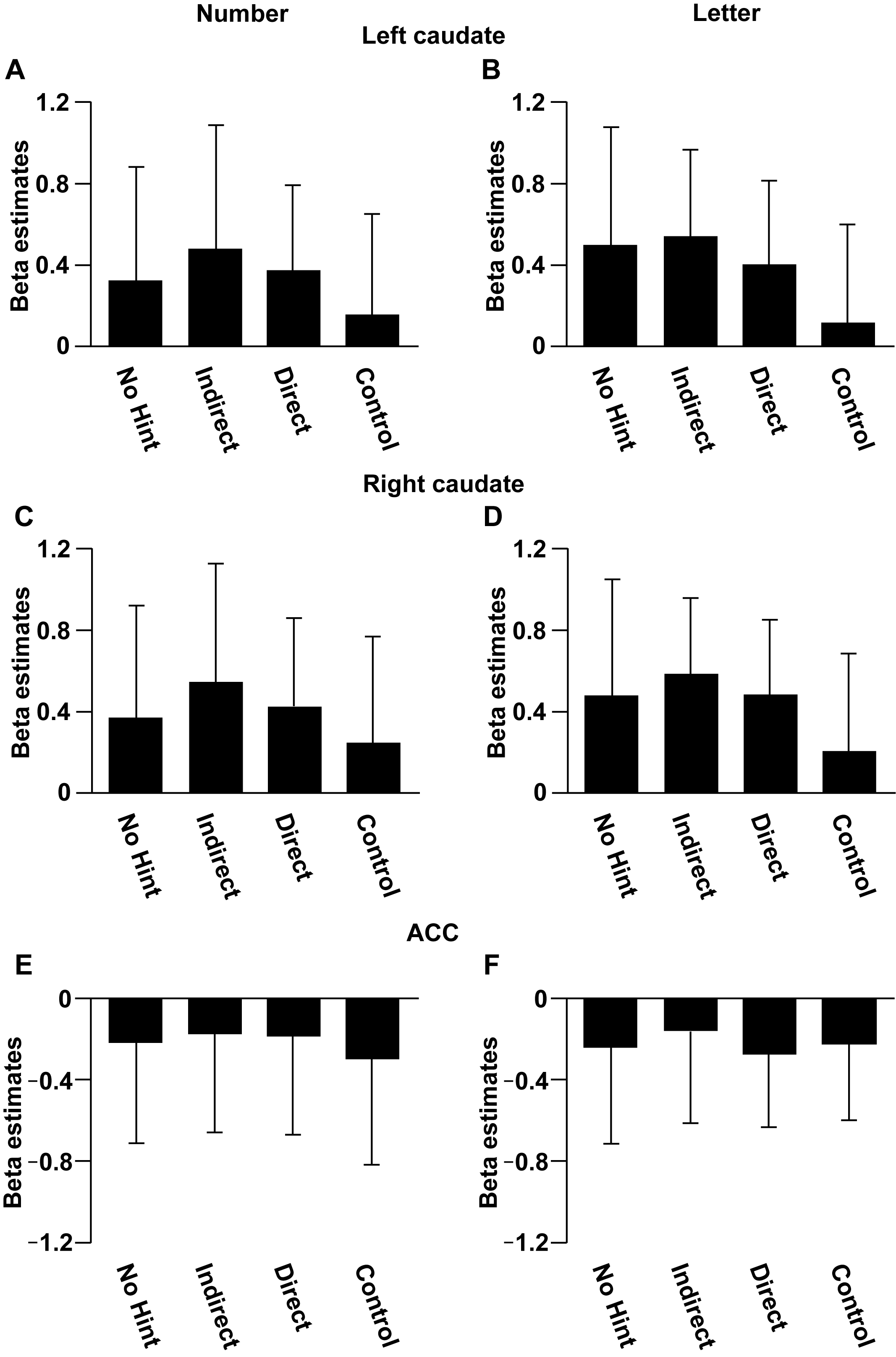

Supplement: S9 Fig — Beta estimates were extracted from the anatomical ROIs of the left caudate (A, B), right caudate (C, D), and ACC (E, F), analysed separately for Number and Letter notations. Error bars, SD. (TIF) [file pone.0168661.s009.tif]

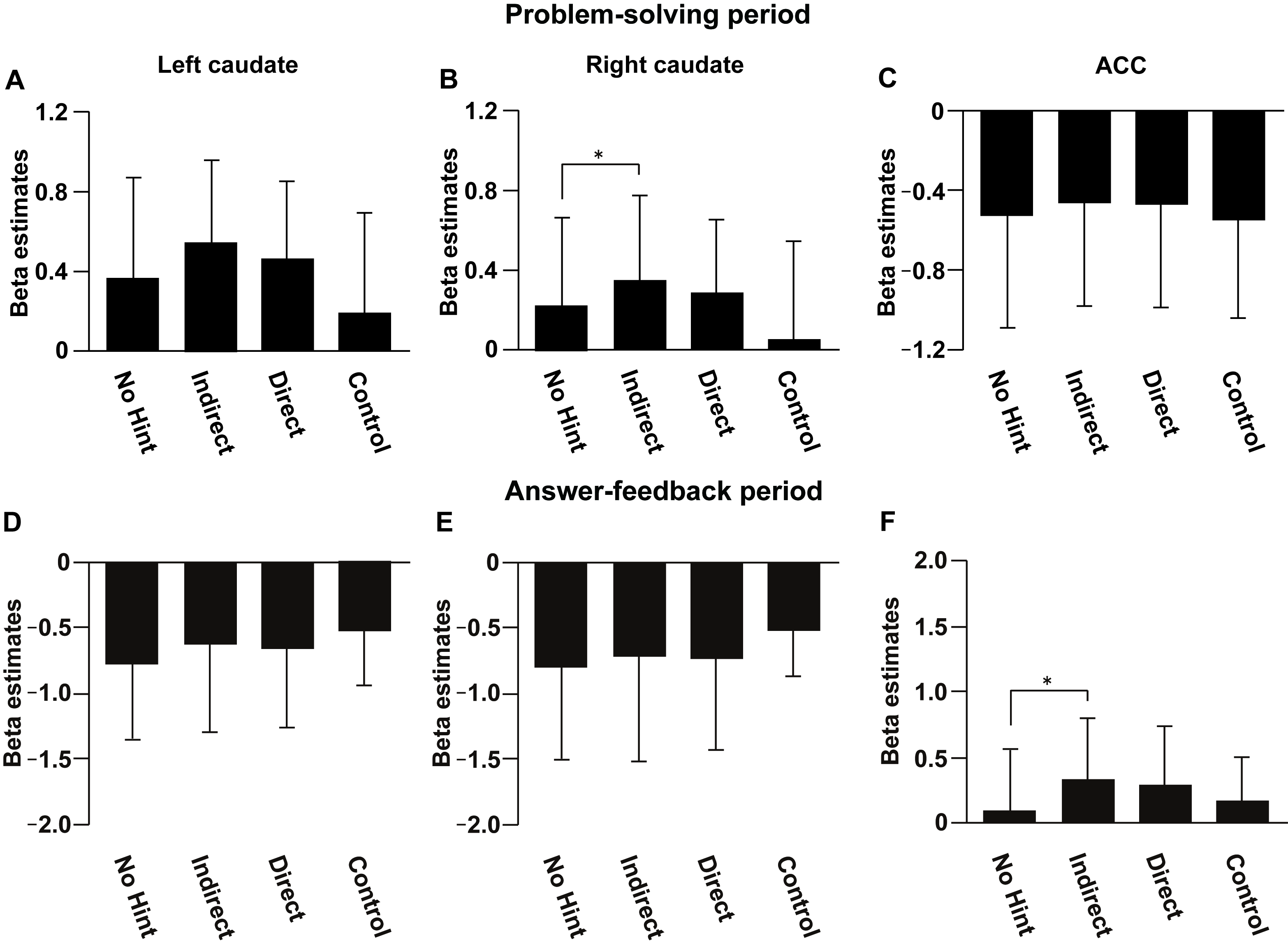

Supplement: S10 Fig — Beta estimates were extracted from the functionally defined ROIs of the left caudate (A, D), right caudate (B, E), and ACC (C, F) for both problem-solving period and answer-feedback period. *P < 0.05. Error bars, SD. (TIF) [file pone.0168661.s010.tif]

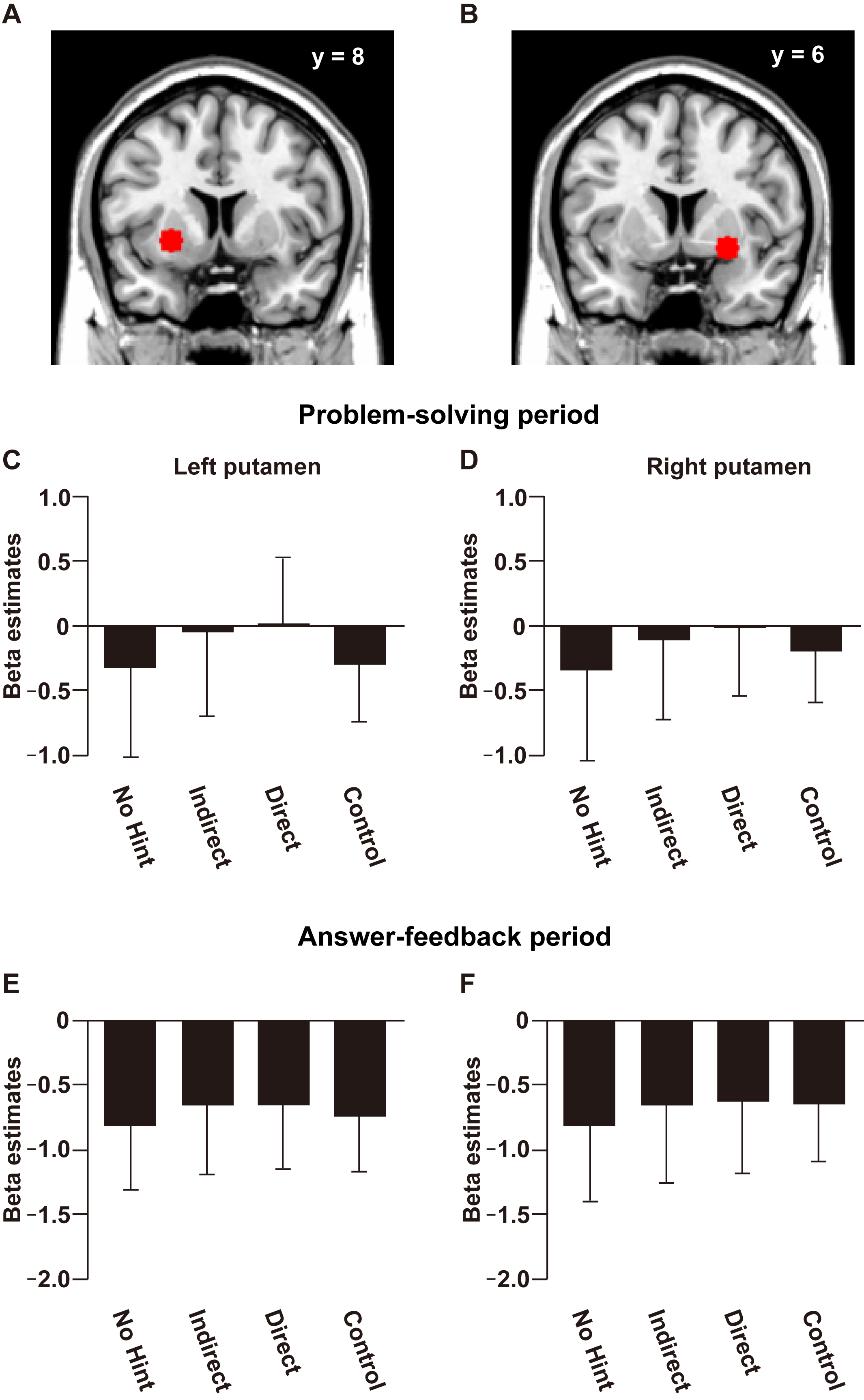

Supplement: S11 Fig — Beta estimates were extracted from the anatomically defined ROIs of the bilateral ventral putamen determined based on the previous study [18]. (TIF) [file pone.0168661.s011.tif]

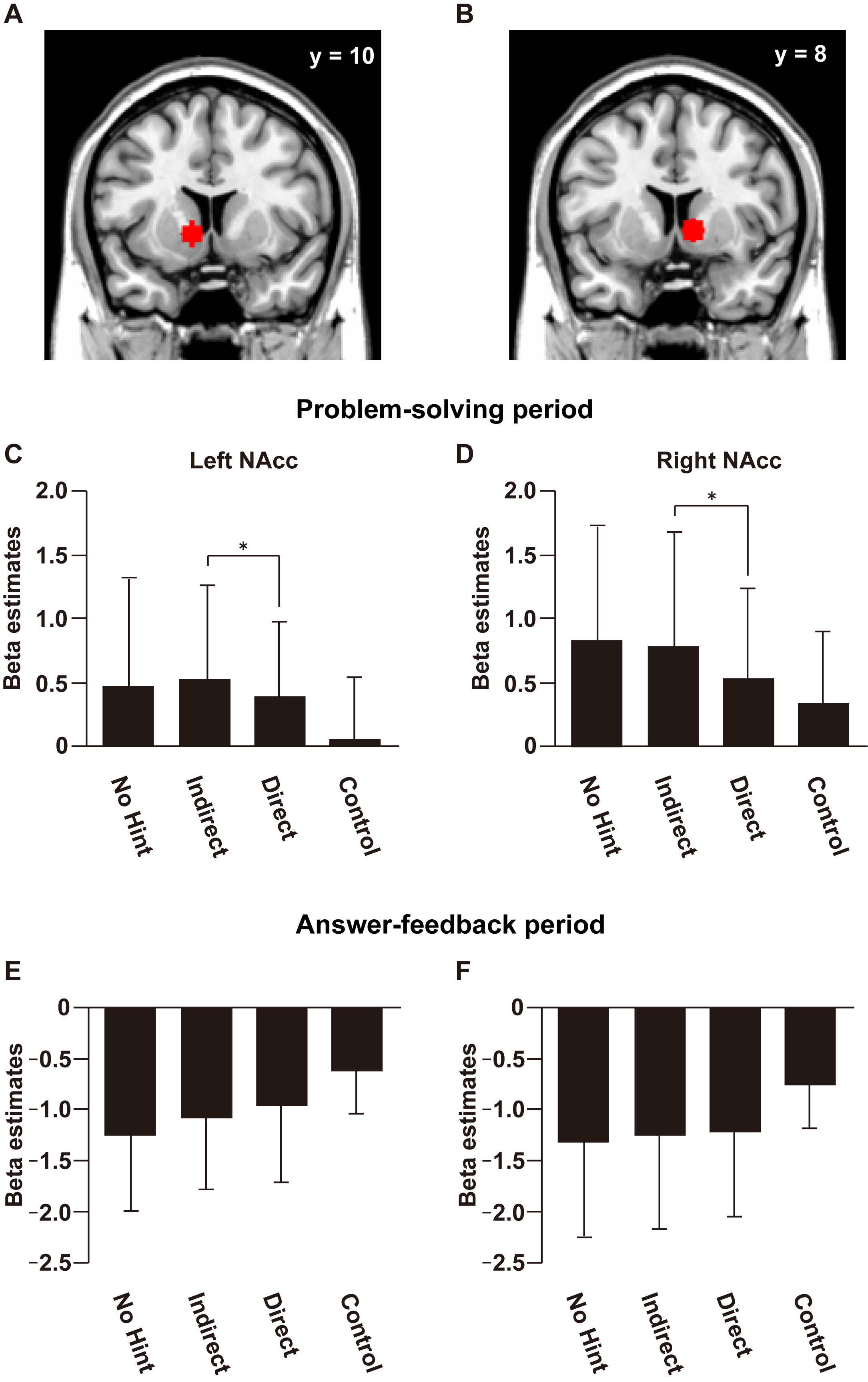

Supplement: S12 Fig — Beta estimates were extracted from the anatomically defined ROIs of the bilateral nucleus accumbens (NAcc) determined based on the previous study [19]. (TIF) [file pone.0168661.s012.tif]

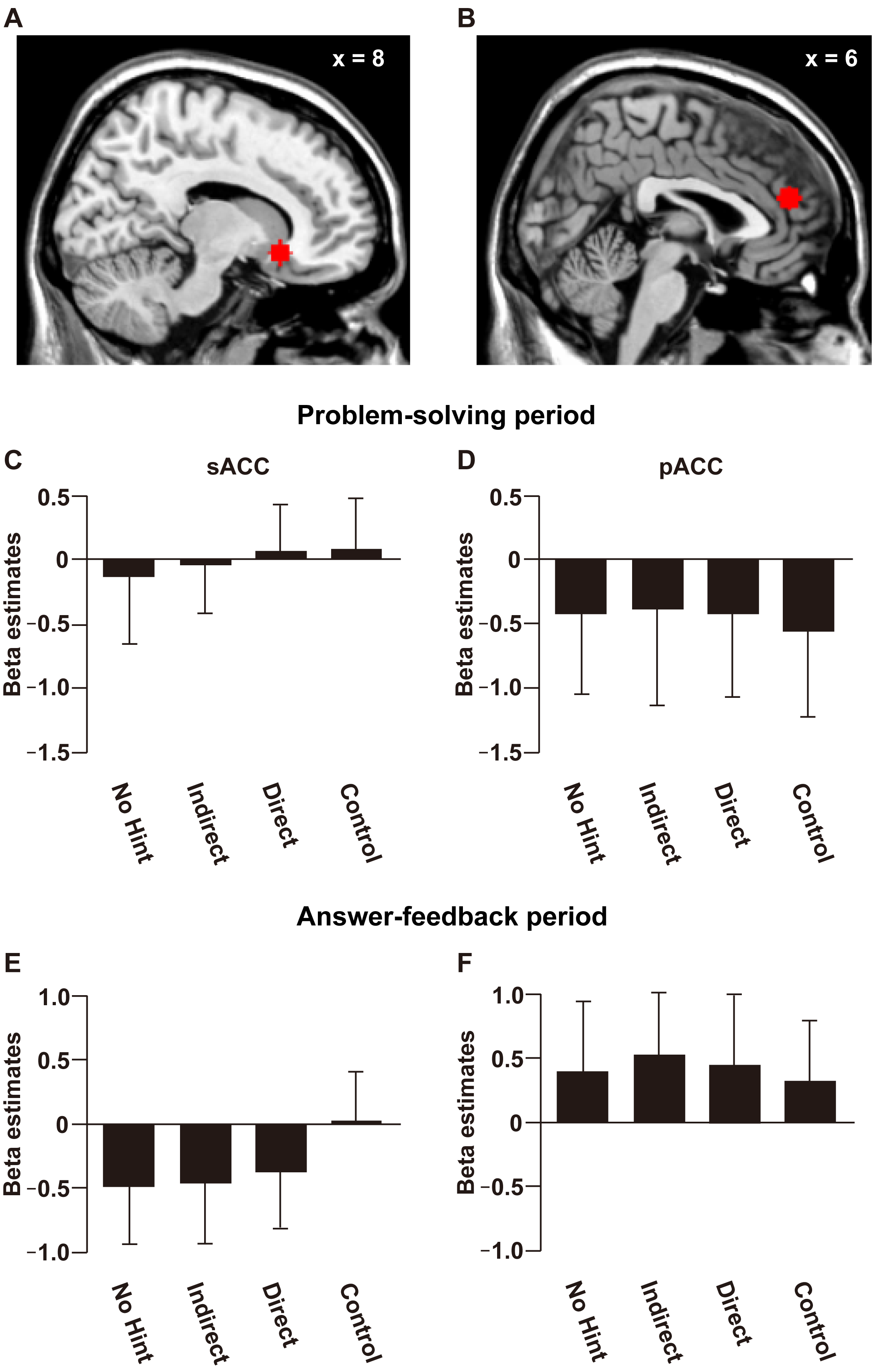

Supplement: S13 Fig — Beta estimates were extracted from the anatomically defined ROIs of the subcallosal ACC (sACC) and pregenual ACC (pACC) determined based on the previous study [27]. (TIF) [file pone.0168661.s013.tif]

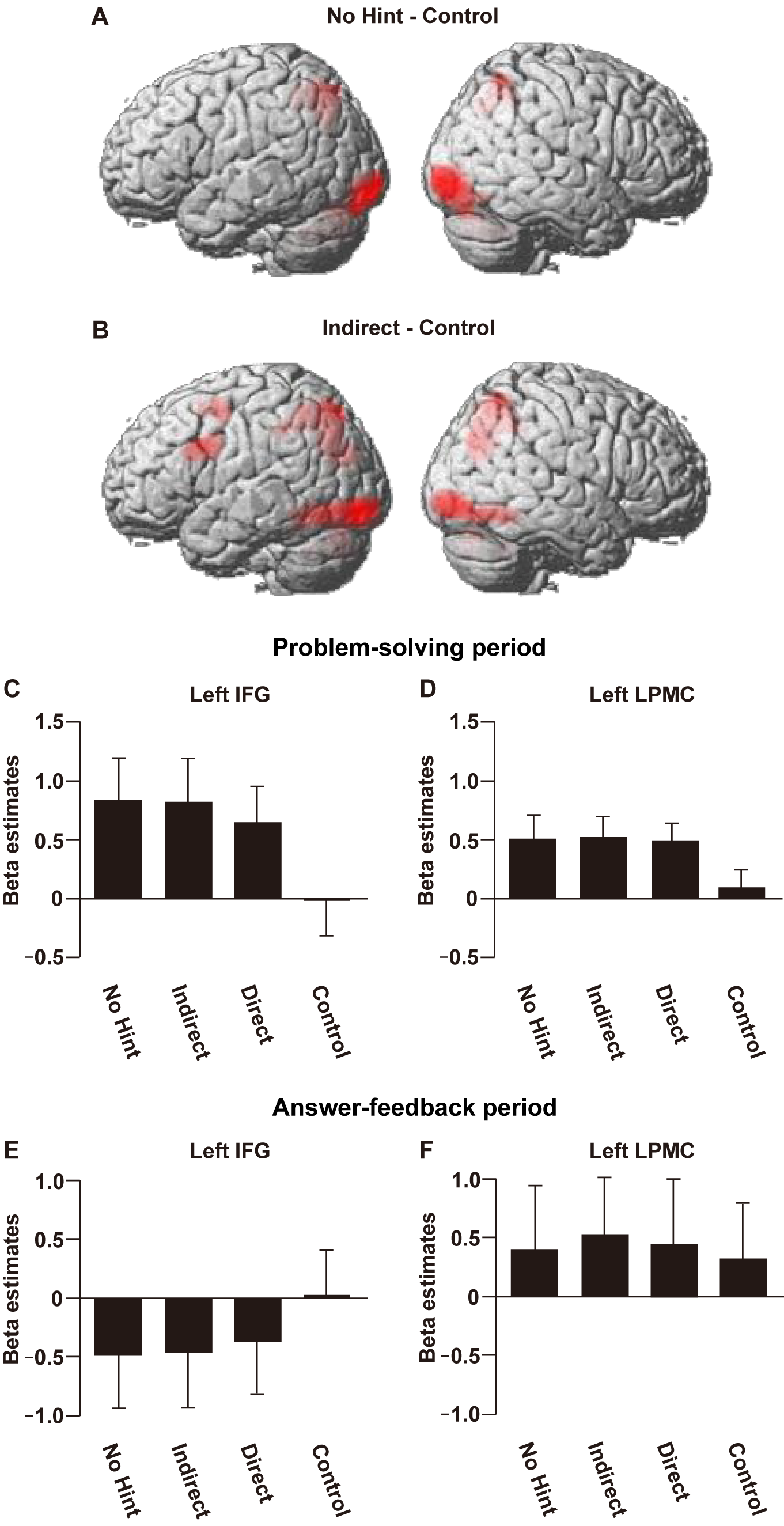

Supplement: S14 Fig — The cortical activation maps of No hint–Control (A) and Indirect–Control (B) were projected onto the standard brain (P < 0.001 for voxel level, P < 0.05 for cluster level, with topological FDR correction). See Table D in S1 File for stereotactic coordinates. Beta estimates for four conditions were extracted from functionally defined ROIs (left IFG and LPMC) of Indirect–Control contrast (C-F). Error bars, SD. (TIF) [file pone.0168661.s014.tif]
