# Supplementary material for: Sense of Accomplishment Is Modulated by a Proper Level of Instruction and Represented in the Brain Reward System
Source: PLoS One. 2017 Jan 4;12(1):e0168661. doi: 10.1371/journal.pone.0168661 (PMC5215289; doi:10.1371/journal.pone.0168661)
Supplement: S1 File — A supporting information file contains the correlation analyses between regressors, the effect of no response trials, analyses of rule-transfer effect, functionally defined region of interest (ROI) analyses, anatomical ROI analyses in the bilateral ventral putamen, anatomical ROI analyses in the bilateral nucleus accumbens (NAcc), anatomical ROI analyses in the ACC subparts, activations without a parametric modulation regressor by SA, and Table A-D. Table A in S1 File. The number of no response trials. The average number of no response trials (mean±SD) are shown for each condition, under each notation. Table B in S1 File. Regions where activations were modulated by SA in the answer-feedback period. Stereotactic coordinates (x, y, z) in the MNI space (mm) are shown for each activation peak of Z-values. dlPFC, dorsolateral prefrontal cortex; PCG, postcentral gyrus. Table C in S1 File. Regions where activations were modulated by SA for the correct trials. Stereotactic coordinates (x, y, z) in the MNI space (mm) are shown for each activation peak of Z-values. SMA, supplementary motor area; dmPFC, dorsomedial prefrontal cortex; PG, parahippocampal gyrus. Table D in S1 File. Direct comparison among instruction levels. Stereotactic coordinates (x, y, z) in the MNI space (mm) are shown for each activation peak of Z-values. IPL, inferior parietal lobule; OP, occipital pole; IFG, inferior frontal gyrus; LPMC, lateral premotor cortex; OTG, occipitotemporal gyrus. The region with an asterisk is included within the same cluster shown one row above. (DOCX) [file pone.0168661.s015.docx]

**Sense of Accomplishment is Modulated by a Proper Level of Instruction and Represented in the Brain Reward System**

Tomoya Nakai, Hironori Nakatani, Chihiro Hosoda, Yulri Nonaka, and Kazuo Okanoya

**1. Correlation analyses between regressors**

To confirm that predicted signals of problem-solving period and answer-feedback period were well-separated, we performed a correlation analysis of regressors. For each participant, we convolved a hemodynamic response function with box-car functions in the problem-solving period and feedback period, and calculated correlation coefficient between these two convolved functions. The average correlation coefficient was –0.20 ± 0.09 (all conditions combined). This low coefficient value suggests that signal overflow effect was limited.

**2. The effect of no response trials**

We examined the number of no response trials in Table A, and we tested the effect of such trials by performing two-way rANOVA (Notation × Condition). This analysis did not show any significant effect (*P* > 0.27). Furthermore, the average SA of no response trials were 1.25 ± 0.42. Considering that those trials were included in Hard trials (which had small SA, Fig 3) in the behavioural analysis, no response trials would not affect the current results.

**3. Analyses of rule-transfer effect**

We examined the rule-transfer effect among conditions, because we used the problems with the same rule under multiple instructions, and knowing a rule under certain instruction (i.e., Indirect) might help participants to find the rule of the given problem under other instruction (i.e., No hint). Besides the transfer effect among trials, we also considered a general learning effect of problem-solving in the current experimental setting, which might be intertwined with the transfer effect. To separate the transfer effect from the general learning effect, we focused on the RT data in the first session. In our experimental design, problems with the same rule always appeared as the pair of No hint and Indirect, or as the pair of Direct and Control. Therefore, we estimated the transfer effect in the following directions: (1) No hint to Indirect, (2) Indirect to No hint, (3) Direct to Control, and (4) Control to Direct (S6 Fig). For example, the transfer effect in the Indirect to No hint direction was evaluated by the RT differences between the No hint condition with initial appearance and that after the Indirect condition. Since participants did not solve problems during the Control condition (and they were given no information about rules), the RT difference in the Control to Direct direction would not reflect the transfer effect, but only the general learning effect within a session. Comparing to the Control to Direct direction, the RT difference of No hint to Indirect direction was not significant (*P* = 0.47), and that in the No hint to Indirect direction was significantly small (*P* < 0.001). Therefore, we found no rule-transfer effect, but only general learning effect in our experimental settings.

**4. Functionally defined region of interest (ROI) analyses**

To confirm that the anatomical ROI analyses reflected the activation contrast related to SA modulation, we also performed the same analyses in the functionally defined ROIs (fROIs) shown in Fig 4. Although the activation difference was significant only between No hint and Indirect conditions in the right caudate (*P* = 0.026), we observed similar activation patterns in these analyses (S10A-C Fig). The fROI analysis in the ACC revealed that the activations under the Indirect condition were significantly larger than those under the No hint condition during the answer-feedback period (*P* = 0.0084, S10F Fig).

**5. Anatomical ROI analyses in the bilateral ventral putamen**

We examined activations in the bilateral ventral putamen, since these regions were also reportedly involved in the reward-based learning [18]. We selected ROIs as a 6 mm sphere from the reported peak coordinates of O’Doherty et al. (2004) (left hemisphere, –26, 8, –4; right hemisphere, 26, 6, –8) [18]. We did not find clear SA-like activation patterns in these regions (S10 Fig).

**6. Anatomical ROI analyses in the bilateral nucleus accumbens (NAcc)**

We investigated activations in the bilateral NAcc, which was reportedly involved in the reward anticipation in the previous meta-analysis study [19]. The results did not show clear SA-like activation patterns (but some tendency in the left NAcc) (S12 Fig).

**7. Anatomical ROI analyses in the ACC subparts**

We extracted signals from subpart of the ACC, because anatomical ACC ROI used in the above analysis includes large areas of cingulate cortex (S8C Fig). We selected ROIs as 6 mm sphere from the reported peak coordinates of Rogers et al. (2003) (pregenual ACC [pACC], 2, 46, 20; subcallosal ACC [sACC], 12, 20, –10) [27]. As a result, we found different activation patterns in the pACC and sACC (S13 Fig).

**8. Activations without a parametric modulation regressor by SA**

In order to know the brain regions generally related to problem-solving activity in our experiment, we examined neural activation without including SA as regressors, and compared activation under each instruction (S14 Fig and Table D). We found significant activation in the bilateral inferior parietal lobule, occipital cortex in the No Hint – Control contrast. In the Indirect – Control contrast, we additionally found activation in the left inferior frontal gyrus (IFG) and left lateral premotor cortex (LPMC). Although no significant activation was found in the Direct – Control contrast, fROI analysis indicate that these left-lateralized regions are generally related to the problem-solving.

|  | Number | Letter |
| --- | --- | --- |
| No hint | 0.19 ± 0.40 | 0.24 ± 0.70 |
| Indirect | 0.10 ± 0.30 | 0 |
| Direct | 0 | 0.05 ± 0.22 |
| Control | 0 | 0.19 ± 0.87 |

**Table A**

**Table B**

| **Brain region** | **BA** | **Side** | ***x*** | ***y*** | ***z*** | ***Z*-Value** | **Voxels** |
| --- | --- | --- | --- | --- | --- | --- | --- |
| vmPFC | 11 | R | 16 | 50 | –4 | 3.1 | 1 |
| dlPFC | 9 | L | –12 | 56 | 34 | 3.5 | 23 |
|  |  |  | –14 | 46 | 48 | 3.3 | 5 |
| ACC | 11 | M | –6 | 34 | 2 | 3.8 | 125 |
|  |  |  | –6 | 56 | 6 | 3.5 | * |
|  |  |  | –2 | 46 | –2 | 3.4 | * |
| PCG | 4 | L | –34 | –24 | 54 | 3.5 | 50 |
| OP | 17 | R | 14 | –88 | 0 | 3.2 | 2 |
| LOG | 19 | R | 14 | –54 | –18 | 3.5 | 14 |

**Table C**

| **Brain region** | **BA** | **Side** | ***x*** | ***y*** | ***z*** | ***Z*-Value** | **Voxels** |
| --- | --- | --- | --- | --- | --- | --- | --- |
| dmPFC | 32 | M | 6 | 34 | 40 | 3.8 | 181 |
|  | 8 | M | 4 | 22 | 48 | 3.6 | * |
| vmPFC | 11 | R | 26 | 38 | –14 | 3.6 | 14 |
| dlPFC | 46 | R | 40 | 54 | 2 | 3.9 | 82 |
| vlPFC | 47 | R | 28 | 32 | –4 | 3.9 | 71 |
| LPMC | 6 | R | 50 | 4 | 50 | 3.5 | 71 |
|  |  |  | 38 | 2 | 56 | 3.3 | * |
| SMA | 6 | M | –4 | 10 | 56 | 3.4 | 41 |
| IFG | 44 | L | –52 | 12 | 40 | 3.3 | 22 |
|  | 44 | R | 52 | 10 | 46 | 3.1 | 1 |
|  | 45 | R | 44 | 32 | 34 | 3.6 | 121 |
|  |  |  | 46 | 32 | 22 | 3.3 | * |
| Insula | 13 | L | –24 | 28 | 4 | 4.1 | 15 |
|  |  |  | –34 | 16 | 8 | 3.8 | 99 |
| Insula |  | R | 42 | 18 | 2 | 3.5 | 35 |
| Caudate |  | R | 8 | 12 | 2 | 4.2 | 77 |
| Thalamus |  | M | 4 | –16 | 10 | 3.5 | 53 |
|  |  | M | –8 | –4 | –2 | 3.2 | 5 |
| PG | 30 | L | –20 | –30 | –10 | 3.3 | 3 |
| ACC | 24 | M | 12 | 36 | 22 | 3.4 | 10 |
| MCC | 23 | M | 0 | –12 | 30 | 3.2 | 1 |
| Precuneus | 7 | L | –14 | –68 | 34 | 3.7 | 22 |
| IPL | 40 | R | 34 | –38 | 36 | 3.3 | 1 |
| LOG | 19 | R | 24 | –58 | 34 | 3.1 | 1 |
|  |  |  | 28 | –80 | 4 | 3.5 | 67 |
| MOG | 18 | R | 18 | –90 | 0 | 3.5 | * |
|  |  | M | –8 | –72 | –24 | 3.3 | 6 |
| OP | 17 | R | 28 | –96 | 10 | 3.4 | 41 |
| Cerebellum |  | L | –36 | –38 | –36 | 3.7 | 27 |

**Table D**

| **Brain region** | **BA** | **Side** | ***x*** | ***y*** | ***z*** | ***Z*-value** | **Voxels** |
| --- | --- | --- | --- | --- | --- | --- | --- |
| **No hint – Control** | | | | | | | |
| IPL | 7 | L | –24 | –62 | 48 | 5.3 | 883 |
|  | 7 | R | 28 | –58 | 52 | 4.2 | 452 |
|  |  |  | 26 | –68 | 44 | 4.0 | * |
| MOG | 18 | L | –20 | –94 | –8 | > 8.0 | 4109 |
|  |  | R | 26 | –90 | –6 | > 8.0 | * |
| OP | 17 | M | –2 | –74 | –24 | 6.0 | * |
| **Indirect – Control** | | | | | | | |
| LPMC | 6 | L | –28 | –2 | 54 | 5.8 | 446 |
| IFG | 44 | L | –42 | 4 | 30 | 5.1 | 702 |
|  |  |  | –54 | 12 | 34 | 3.3 | * |
|  | 45 | L | –38 | 14 | 26 | 4.9 | * |
| IPL | 7 | L | –24 | –62 | 48 | 5.8 | 1846 |
|  |  |  | –16 | –70 | 54 | 4.9 | * |
|  | 7 | R | 28 | –58 | 50 | 4.5 | 1348 |
|  | 40 | R | 26 | –46 | 42 | 4.4 | * |
| LOG | 19 | L | –26 | –68 | 28 | 4.9 | * |
|  | 19 | R | 32 | –72 | 34 | 5.1 | * |
| MOG | 18 | R | 28 | –90 | –6 | 5.7 | 1248 |
| OTG | 37 | R | 44 | –62 | –12 | 4.8 | * |
| MOG | 18 | L | –24 | –92 | –10 | 5.6 | 1644 |
| OTG | 37 | L | –46 | –60 | –10 | 5.4 | * |
| LOG | 19 | L | –38 | –80 | –10 | 5.2 | * |
| Cerebellum |  | M | –4 | –74 | –26 | 5.1 | 843 |
|  |  |  | –2 | –56 | –36 | 4.6 | * |
